# Integrated genomic approaches identify major pathways and upstream regulators in late onset Alzheimer’s disease

**DOI:** 10.1038/srep12393

**Published:** 2015-07-23

**Authors:** Xinzhong Li, Jintao Long, Taigang He, Robert Belshaw, James Scott

**Affiliations:** 1 Centre for Biostatistics, Bioinformatics and Biomarkers, Plymouth University, Plymouth UK; 2 Institute of Cardiovascular and Cell Sciences, St. George University, London UK; 3 School of Biomedicine and Healthcare Sciences, Plymouth University, Plymouth UK; 4 National Heart and Lung Institute, Imperial College, London UK

## Abstract

Previous studies have evaluated gene expression in Alzheimer’s disease (AD) brains to identify mechanistic processes, but have been limited by the size of the datasets studied. Here we have implemented a novel meta-analysis approach to identify differentially expressed genes (DEGs) in published datasets comprising 450 late onset AD (LOAD) brains and 212 controls. We found 3124 DEGs, many of which were highly correlated with Braak stage and cerebral atrophy. Pathway Analysis revealed the most perturbed pathways to be (a) nitric oxide and reactive oxygen species in macrophages (NOROS), (b) NFkB and (c) mitochondrial dysfunction. NOROS was also up-regulated, and mitochondrial dysfunction down-regulated, in healthy ageing subjects. Upstream regulator analysis predicted the TLR4 ligands, STAT3 and NFKBIA, for activated pathways and RICTOR for mitochondrial genes. Protein-protein interaction network analysis emphasised the role of NFKB; identified a key interaction of CLU with complement; and linked TYROBP, TREM2 and DOK3 to modulation of LPS signalling through TLR4 and to phosphatidylinositol metabolism. We suggest that NEUROD6, ZCCHC17, PPEF1 and MANBAL are potentially implicated in LOAD, with predicted links to calcium signalling and protein mannosylation. Our study demonstrates a highly injurious combination of TLR4-mediated NFKB signalling, NOROS inflammatory pathway activation, and mitochondrial dysfunction in LOAD.

Alzheimer’s disease (AD) is a devastating dementia affecting 5–10% of people over 65 years, and 30% of people older than 85 years. AD accounts for around 60 percent of dementia and affects 44 million people globally, 5.2 million in the USA and 850,000 in the UK. In the absence of effective treatments, these numbers are estimated to increase by up to 50% by 2025 both in the USA and UK (http://www.alzheimers.org.uk/)^1^,^2^.

AD develops slowly over many years with the accumulation of characteristic senile (amyloid) plaques and neurofibrillary tangles (NFT) accompanied by neuroinflammation. Ultimately there is a loss of brain cells and synaptic connections as symptoms develop. Certain mutations in APP, PSEN1 and PSEN2 inevitably lead to early-onset AD (EOAD) at around the age of 50 years in five percent of sufferers. Approximately 50% of the risk of late onset AD (LOAD) can be explained by genetic factors. The apolipoprotein E variant, APOE4, found in 15% of the population, is the major genetic risk factor for LOAD. APOE4 contributes around six percent to the phenotypic variance in AD, and it is estimated that between 40% and 65% of people diagnosed with AD have one or two copies of the variant APOE4 gene^1^. Genome-wide association studies (GWAS) coupled with meta-analysis have identified 21 AD genes with low odds ratios, along with more genes at borderline statistical significance for GWAS^3^. The other half of LOAD risk is conferred by modifiable risk factors, which are mostly also risk factors for cardiovascular disease including atherosclerosis, high blood cholesterol, high blood pressure, midlife obesity, diabetes, smoking, physical inactivity and Western diet (http://www.alz.org). While the genetic and modifiable AD risk factors are linked by related mechanisms involving inflammation, cerebral blood flow, lipid metabolism and cell membrane processes^4^, there is a substantial gap in our understanding of the mechanisms underlying AD pathogenesis.

Meta-analysis combines the results of different independent studies, and allows the discovery of patterns of association due to the increased statistical power of larger combined sample sizes. It is widely used in clinical systematic review^5^, genome wide association studies (GWAS)^3^,^6^ and microarray studies^7^,^8^. The use of public databases, meta-analysis and data integration have been extraordinarily valuable especially in investigating complex diseases such as AD. For example, two studies recently identified TYROBP, which modulates TLR4 signalling in brain microglia, as a potential causal regulator in LOAD, and other studies have identified REST as a major modulator in LOAD or discovered multiple genes interacting mechanistically with APOE4^9^,^10^,^11^,^12^.

Here we used a novel statistical approach to meta-analysis of microarray gene expression datasets to discover differentially expressed genes (DEGs) in AD. The main feature of our approach is that it avoids relying solely on those genes for which there are expression data from each constituent study (we call these the common genes). Our study constitutes the largest dataset so far analysed, comprising 450 cases and 212 controls. We also performed pathway and upstream regulator analyses using QIAGEN’s Ingenuity® Pathway Analysis (IPA®, QIAGEN Redwood City, www.qiagen.com/ingenuity) tools, and protein-protein interaction (PPI) network analysis. Our analyses revealed novel genes with potential involvement in AD, and pathways highly perturbed in AD.

## Results

### Differentially expressed genes from meta-analysis

Meta-analysis was conducted on six previous gene expression studies comprising 450 AD and 212 healthy human brain tissue samples from the frontal cortex, including 23530 unique genes shown in Supplementary Table S1 online. After Bonferroni correction (metaPval < 0.05/23530), 3124 differentially expressed genes (DEGs) were identified (1358 up-regulated and 1766 down-regulated). Only 3838 of the 23530 genes (16.3%) were found in all six studies (the common genes) and only 918 (23.9%) of these common genes were identified as DEGs. Our total of 3124 DEGs also include 1582 (50.6%) genes that were found in five studies; 242 (7.7%) in four; 213 (6.8%) in three and 169 (5.4%) in two studies. Clearly, had we only analysed the common genes, we would have missed most of the DEGs (the entire list of 3124 DEGS can be found in Supplementary Table S2 online). We present here the results of an effect size approach in the meta-analysis; an alternate p-value based meta-analysis approach with Bonferroni correction gave very similar results: it identified 3315 DEGs with 3123 overlapping between these two approaches (representing 99.9% and 94.2% of the DEGs identified separately).

We found 2586 out of the 23530 genes were significantly correlated with Braak pathological stage or frontal atrophy in AD patients using data in Zhang *et al.* study^11^, and 1612 of these were identified as DEGs. This demonstrated a high ratio of enrichment (OR = 21.26, 95%CI 19.32 ~ 23.42, p-value < 2.2E-16, Fisher-test). We also observed a high ratio of enrichment in DEGs between gene expression and frontal lobe atrophy (OR = 16.29, 95%CI 14.94 ~ 17.78, p-value < 2.2E-16). Overall, DEGs have 3.39% (p-value < 2.2E-16, t-test) and 5.54% (p-value < 2.2E-16, t-test) increased correlations compared to non-DEGs with Braak stage and frontal atrophy respectively (Supplementary Fig. S1 online). There are twenty top modules identified in gene co-expression regulatory network in AD in the Zhang *et al.* study, half of which are enriched with DEGs identified in this study (Supplementary Table S3 online), including immune and microglia (OR = 6.49, p-value = 4.93E-146, Fisher-test); synaptic transmission and neurons (OR = 3.5, p-value = 8.89E-43); coated vesicle (OR = 4.41, p-value = 4.41E-23); and unfolded protein (OR = 2.29, p-value = 2.06E-15).

The top 30 DEGs identified in this study are shown in Table 1, which includes 12 up-regulated and 18 down-regulated genes. Only 17 of the 30 genes (56.7%) were reported from all six studies and 13 (43.3%) were missed in one study. For all of these 30 DEGs, there is a strong correlation between gene expression and Braak pathological stage or frontal atrophy in AD patients. Overall the up/down effects and positive/negative correlations agree strongly with each other, and are indicative of strong association with AD pathology for each gene. Six of the top seven DEGs are highly correlated with Braak stage (absolute r > 0.7) and are down-regulated, namely NEUROD6, ZCCHC17, PPEF1, MANBAL, BDNF and CRH. We highlight among the top 30 DEGs the following genes: C1QA, DOK3 and NFKBIB, and MS4A6A. DOK3 docks with TYROBP, a microglial regulator in AD, and with TREM2, which has been linked to AD by GWAS. Together these two proteins limit bacterial lipopolysaccaride (LPS) signalling through TLR4^11^,^13^,^14^. NFKBIA is a major pro-inflammatory transcription factor (TF) downstream of TLR4. C1QA is a major complement component and is regulated by NFKB in macrophages^15^. MS4A6A is the only GWAS-discovered AD gene amongst the top 30. In microglia, its expression co-varies with that of TYROBP^11^. The expression of these top DEGs varies across different brain regions in AD (Supplementary Method and Table S4 online). For example ZCCHC17 was consistently down-regulated in four brain regions including the hippocampus, while NEUROD6 was down-regulated only in medial temporal gyrus and superior frontal gyrus; and MS4A6A was not a DEG in any of these six regions in that particular dataset.

The most recent large GWAS meta-analysis of AD cases and controls^3^ identified 21 AD risk genes. Further studies identified two additional AD loci, TRIP4^16^ and PLD3^17^. These 23 genes were included in our initial 23530 gene pool. Seven of them were identified as DEGs with at least two-fold enrichment compared to non-GWAS genes (OR = 2.86, 95%CI 0.99 ~ 7.36, p-value = 2.56E-2, Fisher-test) indicating that genes detected by GWAS are more likely to be DEGs in AD (Supplementary Table S5 online). The non-DEGs found in GWAS were either missed in too many studies (e.g. TXNDC3) or were inconsistent in their direction of regulation across the studies (e.g. BIN1). Expression of the top five differentially expressed genes found by GWAS - MS4A6A, CD2AP, INPP5D, MEF2C and CLU – all have good correlation with Braak stage and cerebral atrophy.

### Pathway analysis

**Figure 1:**
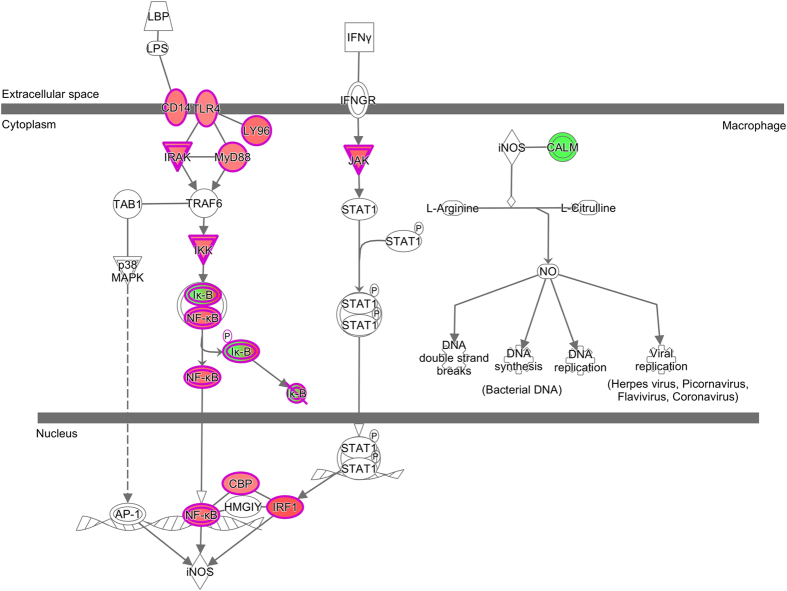
iNOS pathway identified by IPA. The iNOS pathway is identified as one of the significant pathways by IPA. It is also part of NOROS, the most significant pathway. Here all the genes were overlaid to the DEGs. All the up-regulated DEGs are red while CALM is the solitary down-regulated DEG (green). Members of the IK-B family are both up- or down- regulated.

**Figure 2:**
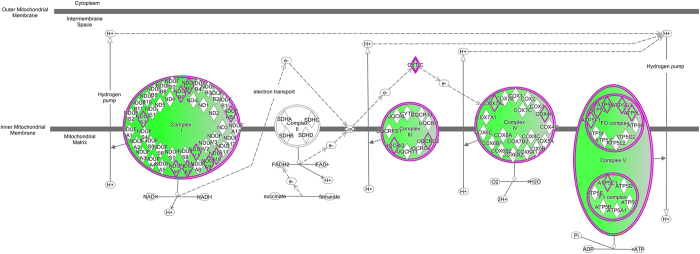
Oxidative Phosphorylation pathway. Though this pathway was identified by IPA for down-regulated DEGs, no up-regulated DEGs are overlaid. The genes in green are suppressed in LOAD.

**Figure 3:**
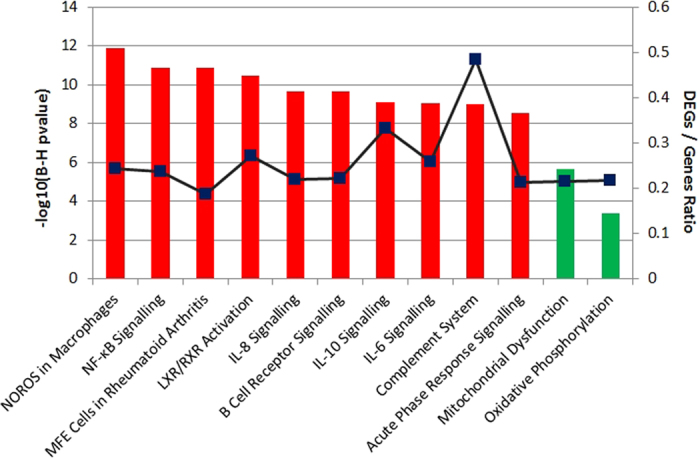
Significant pathways identified by IPA. The top ten significant pathways identified for the up-regulated DEGs (red bar) and two pathways identified for the down-regulated DEGs (green bar). The blue curve shows the ratio between the number of DEGs and the total number of genes in each of these pathways (see the entire list of IPA pathways in Supplementary Table S2 online).

We applied QIAGEN’s Ingenuity® Pathway Analysis (IPA®, QIAGEN Redwood City, www.qiagen.com/ingenuity) tool to our DEGs, analysing the up- and down-regulated DEGs separately. For the 1322 up-regulated DEGs mapped to IPA (genes not mapped to the IPA database were excluded in our pathway analysis), 165 significant canonical pathways were identified (BH adjusted p-value < 0.01, see Supplementary Table S6 online). These include Production of the Nitric Oxide and Reactive Oxygen Species in Macrophages pathway (NOROS, adjPval = 1.26E-12, ratio = 44/180); NFKB Signalling (adjPval = 1.26E-11, ratio = 41/173); the Role of Macrophages, Fibroblasts and Endothelial Cells in Rheumatoid Arthritis (adjPval = 1.26E-11, ratio = 56/298); LXR/RXR Activation (adjPval = 3.16E-11, ratio = 33/121); IL-8 Signalling (adjPval = 2.09E-10, ratio = 40/183) and B Cell Receptor Signalling (adjPval = 2.09E-10, ratio = 39/176). In addition, Interleukin (IL-6, and 10), Complement, PPAR Signalling, Acute Phase Signalling and Toll-Like Receptor Signalling pathways were prominent. In contrast, for 1708 down-regulated DEGs mapped, only three pathways were identified as significant: Mitochondrial Dysfunction (adjPval = 2.24E-06, ratio = 37/172); Oxidative Phosphorylation (adjPval = 4.26E-04, ratio = 24/110) and Aspartate Degradation II (adjPval = 7.24E-3, ratio = 5/7) (see Supplementary Table S6 online). Thus pathways involving TLR signalling, NFKB activation, NOROS, iNOS (also known as NOS2), complement and acute phase responses are identified as up-regulated and mitochondrial function as down-regulated in AD (Figs 1 and 2 and Supplementary Fig. S2 online). The top ten pathways identified by the up-regulated DEGs and the two by down-regulated DEGs are illustrated in Fig. 3. Among the significantly perturbed 168 pathways, RELA is involved in 101 pathways, NFKB1 in 100, NFKB2 in 88, NFKBIA in 62 and STAT3 in 32. These pro-inflammatory transcriptional factors are DEGs and likely to play an important role in AD pathogenesis.

Age is the strongest risk factor for AD; we therefore conducted an IPA pathway analysis on a separate ageing dataset (see Methods) to investigate the shared features between AD and ageing. IPA identified 90 significant canonical pathways for up- or down-regulated DEGs in ageing (Supplementary Table S7 online), 38 of these pathways were shared with pathways in AD. Amongst the top up-regulated pathways were EIF2 signalling (adjPval = 1.05E-10, ratio = 41/169), mTOR signalling (adjPval = 1.58E-5, ratio = 33/181), Integrin signalling (adjPval = 1.66E-5, ratio = 34/194) and STAT3 pathway (adjPval = 6.17E-5, ratio = 18/73). Also active were stem cell, semaphorin and Rho pathways. The top down-regulated pathways included Oxidative Phosphorylation (1.35E-6, ratio = 28/96) and Mitochondrial Dysfunction (adjPval = 1.82E-6 ratio = 37/157). Overall these ageing pathways indicate that the inflammatory pathway is activated, mitochondrial function is suppressed, and regenerative functions are activated.

**Figure 4:**
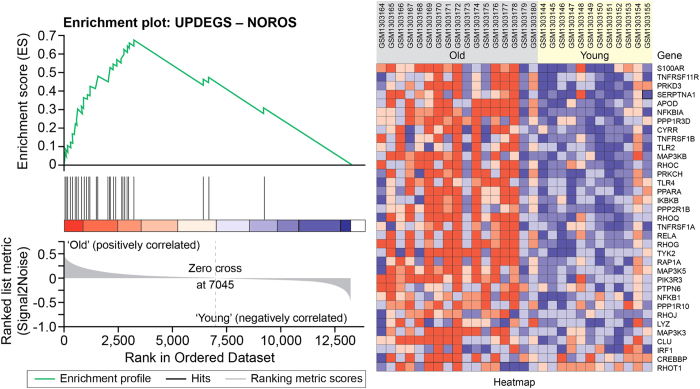
Gene set enrichment analysis (GSEA) for NOROS. GSEA confirms that the NOROS gene set containing up-regulated LOAD DEGs is enriched in old people. Microarray data for ageing (GSE53890) were analysed using GSEA software to identify significant gene sets (see Methods). The enrichment plot on the left shows the distribution of genes in the set that are correlated with the old or young phenotype. The heatmap on the right shows where gene expression is relatively high (red) or low (blue) for each gene in the indicated sample.

The top two pathways in AD, NOROS and NFKB, were not detected in ageing by IPA. We therefore chose to use gene set enrichment analysis (GSEA)^18^,^19^ to analyse in the ageing datasets about the up-regulated AD DEGs in NOROS and NFKB Signalling pathways, and the down-regulated AD DEGs in Mitochondrial Dysfunction (MitoDys) and Oxidative Phosphorylation (OXPHOS) (see Methods). GSEA identified a set of up-regulated gene sets in ageing which included the above NOROS and NFKB sets as second and third most significant gene sets (Fig. 4, Supplementary Fig. S4 and Supplementary Table S8 online). NOROS contains 44 up-regulated AD DEGs, 35 of which were mapped to the ageing dataset and found to be enriched in ageing (nominal p-value < 2.2E-16, FDR < 2.2E-16). 32 of these AD DEGs are also enriched genes in ageing and include the NFKB complex components, NFKBIA, NFKB1 and RELA; the GWAS gene CLU; PPARA; and the TNF receptor superfamily members TNFRSF11B, TNFRSF1A and TNFRSF1B. The NFKB set contains 41 up-regulated AD DEGs, 35 of which were mapped to the ageing dataset with enrichment (nominal p-value < 2.2E-16, FDR < 2.2E-16). GSEA also identified KEGG Oxidative Phosphorylation, Parkinson’s Disease, Huntington’s Disease and Alzheimer’s Disease as the top down-regulated gene sets in ageing, followed by the MitoDys and OXPHOS sets. This result further implicates inflammation through NF-κB, iNOS activation and ROS production in both normal ageing and AD. In addition, NF-κB activation has previously been observed in mouse ageing models^20^ and recently revealed by epigenomics study in mice and humans^21^.

### Upstream transcription regulators

The upstream regulator analysis (URA) tool is a novel function in IPA which can, by analysing linkage to DEGs through coordinated expression, identify potential upstream regulators including transcription factors (TFs) and any gene or small molecule that has been observed experimentally to affect gene expression^22^. It has recently been used to robustly identify repressor element 1-silencing transcription factor (REST) as an important regulator in AD and ageing^12^. For the up-regulated DEGs in AD, IPA identified 230 activated potential upstream regulators (Bonferroni corrected p-value < 0.05, see Supplementary Table S9 online). The top upstream activated regulator is predicted to be LPS, whose target receptor is TLR4. In addition to LPS, multiple other bacterial and endogenous TLR4 ligands, including debris from necrotic cells, connective tissue, coagulation factors and importantly Alzheimer’s amyloid ß peptide (Aß) have been identified^23^. TLR4, STAT3 and NFKBIA are DEGs that are also identified here as important upstream regulators of gene up-regulation. Both STAT3 and NFKBIA are activated through the adaptor protein MYD88, which is a downstream signalling gene for TLRs^24^ and itself is a DEG. STAT3 has a major role in promoting inflammatory pathways. The mechanistic networks for these regulators generated by IPA are demonstrated in Supplementary Fig. S3 online. Those upstream regulators include 33 activated TFs, 11 of which are DEGs with nine up-regulated and two down-regulated. The differentially expressed TFs include 3 NFKB components. Other upstream TFs that are DEGs are (a) SMAD4, which is downstream of the growth factor TGFB, and is a major determinant of the pathway Diseases and Biofunctions, Connective Tissue Disorders; (b) IRF1 and IRF7, which both regulate the transcription of interferon genes and are linked to TLR signalling; (c) CEBPB, which is important in the regulation of genes such as interferons that are involved in immune and inflammatory responses, acute-phase and cytokine genes, as well as connective tissue genes. Together these observations re-emphasise the importance of inflammatory and connective tissue responses in AD.

Among the down-regulated DEGs, IPA identified the AD-associated TF REST (adjPval = 1.53E-9) as an upstream regulator, regulating 27 down-regulated DEGs. RICTOR is identified as another activated upstream regulator (adjPval = 4.69E-3), and its activation is predicted to regulate 36 down-regulated DEGs including multiple mitochondrial genes (see Supplementary Tables S9 and S10 online).

We applied URA to ageing as well. Relevant upstream regulators are shown in Supplementary Table S11 online. REST (adjPval = 2.78E-3) is identified as an upstream regulator in ageing as previously described^12^ and regulates 24 targets. RICTOR is also detected as another upstream regulator (adjPval = 6.35E-6) and regulates 46 targets in ageing.

### Protein -protein interaction (PPI) networks

**Figure 5:**
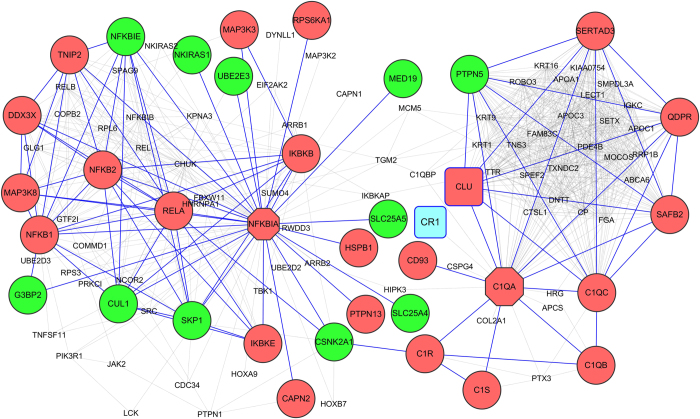
Protein-protein interaction (PPI) network for NFKBIA, C1QA and CLU. The red nodes indicate the up-regulated DEGs, while green nodes indicate the down-regulated DEGs. Grey and blue nodes are not DEGs. All the DEGs are linked by blue lines. NFKBIA, C1QA and CLU are among the top 30 DEGs (see Table 1). Both CLU and CR1 are GWAS genes (see Supplementary Table S2 online). This is a subnetwork of the PPI network for the top 30 DEGs (see Supplementary Fig. S5 online).

To further elucidate the interactions of the top DEGs and the GWAS findings we performed human PPI network analysis (see Methods). We constructed a subnetwork by mapping the top 30 DEGs to the PPI network (16 DEGs mapped) and extracted their first neighbour nodes (FNN) from the PPI network. This subnetwork contains 175 nodes and 998 edges including three GWAS discovered genes: CLU, CR1 and INPP5D (Supplementary Fig. S5 online). The two top hubs were NFKBIA and CLU (see Supplementary Table S12 online). NFKBIA is connected to 64 genes in this subnetwork, including 13 up-regulated DEGs and 10 down-regulated DEGs. These include the NFKB complex up-regulated DEGs RELA, NFKB1, NFKB2 and IKBKE; the down-regulated NFKB complex gene NFKBIE; and unperturbed genes CHUK, EIF2K2, IKBKAP, RELB, REL and NFKBIB. CLU links to 32 genes including five DEGs: C1QA, C1QC, QDPR, SERTAD3 and SAFB2. C1QA is linked to the complement component GWAS discovered gene CR1, which is not a DEG, and other complement DEGs C1QB, C1R and C1S (Fig. 5, a subnetwork of Supplementary Fig. S5). Although the function of CLU is not known, the linkages identified suggest a key role in innate immune responses through complement activation. Thus the PPI overwhelmingly identifies the importance of NFKB in AD.

We were able to map 14 of the 23 AD GWAS findings to the human PPI network. MS4A6A is the only DEG amongst these that is not included. The resulting FNN subnetwork contained 332 nodes and 7692 edges (Supplementary Fig. S6 online). There was an average of 23 neighbours per node, which was much higher than that of the whole human PPI network (OR = 5.22, p-value < 2.2E-16, Fisher-test). This subnetwork included 78 DEGs with enrichment (OR = 1.44, p-value = 8.30E-3), such as DOK3, C1QA, C1QC, GFAP, SYK, STAT3, SNCA and TYROBP. APOE is the top hub (124 first neighbours) and links to 18 DEGs; 10 of these are up-regulated and implicated in inflammation (HP, ITGB5, SERPING1, CFB, CFH), lipid metabolism (CHKB, PLTP, DGKG), cytoskeleton (GFAP) or gene expression (NCOA3); eight are down-regulated and implicated in the cytoskeleton (TLN2, KRT5, MAPT, NEFM) endocytosis and lipid metabolism (VLDLR, EXOC6, TTPAL) or DNA repair (DDB1). Of these, PLTP was reported to reduce phosphorylation of tau (MAPT) in human neuronal cells^25^, and is activated through the top pathway, LXR/RXR Activation. Phospholipids have also been described to be important biomarkers of pre-symptomatic AD. PTKB2 is the second top hub linking directly other 55 genes including 13 up-regulated and three down-regulated DEGs. ApoE4 and PTK2B have been previously linked mechanistically to Aß production by ApoE4 stimulated neuroblastoma cells^9^. PTK2B physically interacts with the receptor tyrosine kinase EPHA1, encoded by another AD gene; both of these genes are implicated in vascular inflammation and blood flow. TYROBP and TREM2 are first neighbours in the network. Other hubs are shown in Supplementary Table S13 online.

## Discussion

In this study, we used an approach to meta-analysis in which, by focusing on the prefrontal cortex, all the genes across different studies are included. Previous integrative AD studies either pooled the raw data^9^ or combine the results from different brain regions^26^. Most microarray meta-analysis only use genes reported across all the studies (the ‘common genes’)^26^, so the more the studies that are included in a meta-analysis, the smaller the number of common genes. In our study, to have ignored the many genes that are not common (19692 out of 23530) would have created many false negatives. We would have not only missed important GWAS genes (*CD2AP*, *INPP5D*, *TREM2*, *CLU*, *ABCA7*) but also other genes with known functions in AD such as BDNF^27^. Q-Q plot demonstrates that the normality of the combined metaZscore is improved by including non-common genes. In this respect, our alternate p-value based method performed similarly to our main effect-size based approach (Supplementary Fig. S7 online).

**Figure 6:**
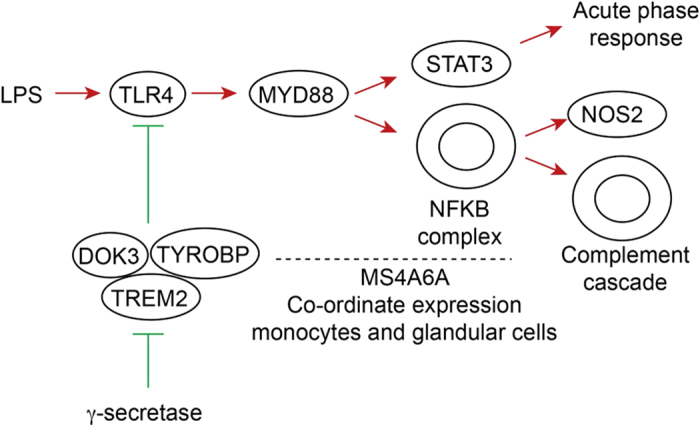
Regulation pathway of LPS/TLR4. The diagram shows the activation of the TLR4 signalling pathway by LPS or by surrogate ligands such as Aß, and activation of STAT3 and NFKB through the adaptor MYD88. NFKB and STAT3 activate NOS2 (iNOS), acute phase responses, complement and other inflammatory processes. The DOK3, TYROBP, TREM2 complex regulates the interaction between LPS and TLR4 and is in turn modulated by У-secretase.

Overwhelmingly our results bring together enhanced TLR4 signalling and activation of NFKB transcription with up-regulation of NO and ROS production and complement as key mechanisms of neuroinflammation in AD (Figs 1 and 6, Supplementary Fig. S3 online). Upstream regulator analysis by IPA identifies the bacterial product LPS as the top upstream regulator. While low levels of LPS are found in human blood, multiple other pathogen-derived and endogenous ligands for TLR4 have been identified, and these include debris from necrotic cells, connective tissue, coagulation factors and importantly AD ß-amyloid peptide^23^,^28^. Amongst the DEGs recovered in our IPA and PPI analysis, NFKB is highlighted by our study as a key pro-inflammatory TF in AD. NFKB activates NOS2 in microglia^29^,^30^, and NOROS activation is identified as the top pathway by IPA. LPS is also a key regulator of the production of ROS by NADPH oxidase as identified in the NOROS pathway. The combined activation of NO and ROS production could stimulate creation of highly injurious nitrosative stress, which is particularly damaging to mitochondria^31^. Astrocytes and microglia are also the likely source of complement^32^. We also find evidence for activation of multiple other inflammatory and connective tissue-disorder pathways mediated through pro-inflammatory TF genes such as STAT3, IRF1 and IRF7, CEBPB and SMAD4.

A gene set of up-regulated DEGs in NOROS showed highly significant association with normal ageing. Previously, NFKB, REST and complement C1Q have been associated with normal ageing, but to the best of our knowledge TLR4 signalling and NOROS activation have not^12^,^15^,^20^. Although we did not identify NOROS and NFKB pathways in ageing directly by IPA, GSEA reveals that gene sets with up-regulated AD DEGs in NOROS and NFKB were highly activated in ageing samples (Fig. 4 and Supplementary Fig. S4 online). These results suggest that AD might develop through direct corruption of the normal ageing process, as well as through independent pathways of amyliod plaque and NFT production triggered by ageing. The activation of stem cells, semaphoring and Rho pathways suggest that regenerative functions are activated. The importance of such pathways in normal ageing is highlighted by recent parabiotic studies comparing young and old mice^33^,^34^.

Both DOK3 and TYROBP are identified as up-regulated DEGs. DOK3 and TYROBP are adaptor proteins that modulate the signalling of LPS through TLR4^13^. TYROBP is also a signalling adaptor for TREM2, which is an up-regulated DEG and also a risk gene discovered by GWAS^14^. TYROBP, DOK3 and TREM2 inhibit LPS signalling through TLR4 in macrophages and prevent inflammation^35^. We find that DOK3 also interacts with the INPP5D, which is encoded by an AD risk gene, thereby interfering with phosphatidylinositol metabolism. INPP5D also functions as a negative regulator of inflammatory cell proliferation and survival^36^. Together these results strongly implicate the TLR4 signalling pathway in symptomatic AD.

With regard to the GWAS discovered genes we show that APOE, the major genetic determinant of LOAD, and PTKB2 are the two top hubs in our PPI analysis. While its function is not known, MS4A6A is co-ordinately expressed with TYROBP and complement genes, and is expressed in monocytes and glandular cells (http://www.biogps.org). This suggests a role in the key up-regulated processes we describe, namely TLR4 signalling and complement activation in microglia, and perhaps secretion (Fig. 6). While the newly discovered AD risk gene TRIP4 is not a DEG in our study, it forms a PPI with six DEGs: NFKB1, RELA, CREBBP, ESR1, NCOA1 and TBP. TRIP4 is highly expressed in the immune system and is a target of NFKB^16^.

We discovered a down-regulated series of most highly differentially expressed genes (DEGs), NEUROD6, ZCCHC17, PPEF1, MANBAL, and BDNF, which were strongly correlated with neuropathology. NEUROD6 encodes a TF and its expression is maintained in differentiated neurons in the adult brain, where high levels of its mRNA are sustained in mature pyramidal neurones of the hippocampus, cerebellum and cortical regions associated with learning and memory^37^,^38^,^39^. NEUROD6 links neuronal differentiation to survival and endows the cells with increased oxidative stress tolerance via a network of molecular chaperones, organisation of the cytoskeleton, and enhanced mitochondrial biogenesis and bioenergetics. In mouse, expression of NeuroD6 parallels neuronal differentiation, and directs post mitotic cell fate^40^. A further recent report has inferred that NEUROD6 might be a possible down-regulated biomarker for AD through a comparison of two publicly accessible RNA sequencing (RNAseq) datasets and three microarray datasets^41^. ZCCHC17 (zinc finger, CCHC domain-containing 17), is a novel TF. The CCHC-type zinc finger motif is involved in DNA and RNA binding, and can mediate protein-protein interactions. The function of ZCCHC17 is not known, but it is highly expressed in the brain and in myeloid cells. Another family member, ZCCHC9, can suppress the transcription activity of NFKB, and may play a role in the MAPK signalling pathway^42^. PPEF1 is a serine/threonine-protein phosphatase with two calcium-binding EF-hands and is suggested to be involved in sensory neuron function and development, sharing high sequence similarity with the Drosophila retinal degeneration C (rdgC) gene. MANBAL is a mannosidase, beta A, lysosomal-like gene. Importantly the targeting of proteins to lysosomes and endosomes through the mannose-6-phosphate receptor facilitates the conversion of APP to Aß by the APP processing enzyme BACE1 through co-localisation with APP in early endosomes^43^. BDNF is a well-characterised secreted neurotrophic factor, which supports neuronal survival and the growth of new neurones and synapses^44^. It is active in the hippocampus, cerebral cortex, and basal forebrain—all areas vital to learning, memory, and higher thinking. BDNF levels are reduced in the brain tissues of people with AD, where hippocampus damage lowers the levels of BDNF. BDNF also has a protective role against ß-amyloid toxicity. BDNF gene transcription is regulated by calcium, like NEUROD6 and calcium metabolism is well known to be aberrant in AD. However, the roles of these top DEGs in AD are still not clear.

We see dramatic evidence for mitochondrial dysfunction, notably oxidative phosphorylation, in our AD dataset as well as in ageing. Upstream regulator analysis in IPA identifies RICTOR as a potential regulator of the transcription of multiple genes involved in oxidative phosphorylation and other mitochondrial functions. RICTOR is part of the mTOR pathway, mTORC2 complex, and potentially inhibits nuclear mitochondrial expression through down-regulation of the transcript factor YY1 and transcriptional co-repressor PGC1A^45^,^46^, however, YY1 and PGC1A are not DEGs in AD.

A limitation of our study is that we chose to analyse data from the frontal lobe region in order to maximise the number of directly comparable samples. Unlike the hippocampus and entorhinal cortex that are affected early in AD, as seen in early Braak stage, the frontal lobes are affected later, when disease pathology becomes more pervasive. Nonetheless, similarities in gene expression in AD patients in the frontal lobe region and other brain regions are well documented^47^. In future meta-analysis we propose to integrate data from all brain regions to find globally important genes and pathways.

In conclusion, our meta-analysis strategy maximises the use of AD database information and has correlated DEGs with cerebral pathology. Analysis of the results identifies some well-recognised genes and some new genes in AD. In particular we highlight the possible role TLR4 in AD through (a) its up-regulation, (b) its modulation by TYROBP, DOK3, TREM2 and INPP5D, and (c) its signalling through NFKB leading to microglial NO and complement activation. We confirmed REST as a major predicted upstream regulator in AD and ageing through perturbation of Wnt signalling^12^. We discovered new AD genes, of which the most important are perhaps the strongly down-regulated DEGs NEUROD6, ZCCHC17, PPEF1 and MANBAL. We also present evidence for links between AD and phosphatidylinositol and calcium signalling as well as through the previously known top LOAD genes APOE and PTK2B, which are potentially linked to blood brain barrier dysfunction and blood flow. These insights suggest several new avenues for mechanistic research into AD and potential drug targets.

## Materials and Methods

Our approach to integrating public gene expression data generated by different microarray platforms was as follows. First, we calculated study-specific statistics by comparing mRNA expression level between LOAD and control post-mortem brain tissues in the frontal cortex region by the limma^48^ R package. We then applied an effect size-based meta-analysis method to calculate the combined effect size, and obtained the relevant statistical p-value by assuming a normal distribution with Bonferroni correction for multiple testing to identify combined-study DEGs. Thereafter, we performed pathway and upstream analysis on these DEGs, and constructed networks based on known protein-protein interactions to reveal the relationships at the protein level and thereby identify pathways involving multiple DEGs.

### Data collection and pre-processing

We searched arrayExpress and GEO for all LOAD-related mRNA expression studies that included human post mortem brain tissues from super frontal gyrus (SFG) or prefrontal cortex (PFC), both of which are part of the frontal lobe. We found and downloaded six profile datasets with GEO accession numbers GSE5281, GSE48350, GSE36980, GSE15222, GSE44770 and GSE33000. We also downloaded the GSE53890 human brain ageing dataset for comparison. Please see Supplementary Information for details about data processing.

### Meta-analysis

The basic theory of meta-analysis of microarray study was described initially by Choi *et al.*^49^, and has subsequently been modified and widely applied^7^,^50^,^51^,^52^. Here we briefly provide the application formulas proposed by Marot *et al.*^52^, which involve an inverse variance-weighted effect size meta-analysis method based on moderated *t-*statistics calculated by the limma R package^48^. In a case-control study, for a given gene, assume there are *n*_1_ replicates in the case group and *n*_2_ replicates in the control group. The biased effect size *d* can be estimated by the relevant *t*-statistic, *e.g*., 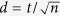 and the biased variance of effect size is given by the following formula:


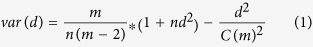


Here 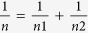 and *m* is the number of degree of freedom which is the sum of the prior degree of freedom and the residual degree of freedom for the linear model of a certain gene. This is the total number of degrees of freedom as calculated by the limma method. The value of C(m) is derived as follows:


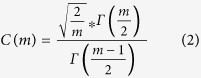


Where *Γ* can be performed by the *gamma* function in R, and therefore the unbiased effect size can be modified as *d*′ = *C*(*m*)*d* . There are two steps to estimate the unbiased variance: first substitute *d*′ into Equation (1) to get the modified variance, then multiply the squared value obtained from equation (2), i.e., *C*(*m*)^2^, to obtain the estimated unbiased variance *var*(*d*′) of the unbiased effect size *d*′. The weight for effect size *d*′ is given by *w* = 1/*var*(*d*′), and the final combined *metaZscore* can be calculated as follows:


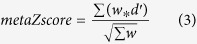


Assuming a normal distribution, the relevant *p-value* can be calculated (denoted as metaPval) in R using *metaPval*=2*(1 − *pnorm*(*abs*(*metaZscore*))). If a gene is missing in a particular sample from a particular study (null value), then the actual sample size will be used in equation (1) or we impute the null values using the impute R package^53^ first. We use the actual sample size method. If a gene is not included in a particular study, then we can either impute its effect size^54^ or simply ignore its contribution^55^ in equation (3). e.g. setting the relevant weight *w* to zero. We adopt the latter method which is also widely applied in GWAS meta-analysis^3^,^6^,^56^,^57^. If a gene is a singleton, i.e. only present in one study, then its Benjamini & Hochberg (BH) adjusted p-value as calculated by the limma would be treated as the final metaPval and the relevant metaZscore would be estimated by a standard normal distribution. Marot *et al.*^52^ also proposed an alternative relatively simple meta-analysis solution, e.g. sample-size weighted one-side p-value combination method. This method substitutes 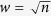 and *d*′ = *ϕ*^−1^(1 − *pval*) into equation (3), where *ϕ*^−1^(*x*) is the inverse cumulative distribution function of standard normal distribution. The effect direction in each study for each gene is recorded. For example, in the final meta-analysis the effect “++?−++” for a certain gene indicates that this gene was up-regulated (+) in the first two and last two studies; down-regulated (−) in the fourth study and missed (?) in the third study. We adjust p-values by Bonferroni correction for stringency and conduct Q-Q plot to test the normality. All calculations were completed in R (http://www.bioconductor.org) and a relevant R package is under development by the authors. We note that, existing microarray gene expression meta-analysis R packages, such as metaMA^52^, assume that the data are from the same genes in each individual study and apply fixed sample size for all genes in the individual studies, thereby ignoring the effect of missing values. In contrast, our approach does not require the same gene dimension for each study, i.e. we work on the combined gene set from all the studies involved in the meta-analysis.

### Identification of activated transcriptional regulator and pathway analysis

We used the commercial QIAGEN’s Ingenuity® Pathway Analysis (IPA®, QIAGEN Redwood City, www.qiagen.com/ingenuity) software for functional pathway and upstream regulatory analysis (URA) of DEGs identified in this study. We used the Benjamini-Hochberg method to adjust canonical pathway p-values, and set 0.01 as the significant threshold. For URA, we used the Bonferroni method to correct the detection p-value and set 0.05 as the significant threshold. Geneset enrichment analysis (GSEA)^18^,^19^ is widely applied to determine whether a predefined geneset shows statistically significant difference between two biological states among a list of genesets. We applied GSEA to the ageing dataset in order to compare four genesets identified from AD meta-analysis (NOROS and NF-kB Signalling pathways, Mitochondrial Dysfunction (MitoDys) and Oxidative Phosphorylation (OXPHOS) and other KEGG (Kyoto Encyclopedia of Genes and Genomes) pathways downloaded from MiSigDB (http://www.broadinstitute.org/gsea/msigdb/). We used phenotype permutation for 1000 times, and chose the weighted signal to noise statistical approach to rank genes and complete the GSEA analysis.

### Protein-protein interaction network analysis

In order to reveal the interactive relationships among the DEGs at the protein level, we first downloaded the human protein-protein interaction network (PPIN) from the Human Protein Reference Database (HPRD, release 9, www.hprd.org), and created a whole human PPIN which contained 8,603 unique protein entries (nodes) and 44,376 unique undirected interactions (edges). This was visualized using Cytoscape 3.0^58^. We then mapped both the DEGs identified by our meta-analysis and known loci of AD risk to this PPIN to build relevant subnetworks.

## Additional Information

**How to cite this article**: Li, X. *et al.* Integrated genomic approaches identify major pathways and upstream regulators in late onset Alzheimer's disease. *Sci. Rep.* **5**, 12393; doi: 10.1038/srep12393 (2015).

## Supplementary Material

Supplementary Information

Supplementary Table S2

## Figures and Tables

**Table 1:** The top most significant differentially expressed genes in our meta-analysis.

Table 1. The top 30 most significant DEGs								
EntrezGene	Symbol	metaZscore	metaPval	avgFC$	Effect#	adjPval	Braak*	Atrophy*
63974	NEUROD6	−9.93	0	0.60	−−−−−−	0	−0.77	−0.62
51538	ZCCHC17	−8.91	0	0.81	−−−−−−	0	−0.73	−0.59
5475	PPEF1	−8.9	0	0.61	−−−−−−	0	−0.76	−0.61
712	C1QA	8.76	0	1.59	?+++++	0	0.61	0.47
63905	MANBAL	−8.76	0	0.87	−−?−−−	0	−0.73	−0.58
627	BDNF	−8.74	0	0.61	−−?−−−	0	−0.76	−0.58
1392	CRH	−8.73	0	0.54	−−?−−−	0	−0.77	−0.58
3707	ITPKB	8.68	0	1.80	++++++	0	0.75	0.57
388341	FAM211A	−8.68	0	0.84	−−?−−−	0		
2289	FKBP5	8.59	0	1.56	++++++	0	0.75	0.58
64231	MS4A6A	8.57	0	1.43	++++++	0	0.62	0.51
78991	PCYOX1L	−8.57	0	0.67	−−−−−−	0	−0.64	−0.59
1846	DUSP4	−8.51	0	0.70	−−?−−−	0	−0.74	−0.54
7108	TM7SF2	−8.49	0	0.78	−−?−−−	0	−0.71	−0.57
6405	SEMA3F	8.47	0	1.32	++++++	0	0.6	0.48
9322	TRIP10	8.42	0	1.38	++++++	0	0.69	0.56
10184	LHFPL2	8.4	0	1.32	++++++	0	0.66	0.56
9315	NREP	−8.39	0	0.75	−−−−−−	0		
381	ARF5	−8.35	0	0.78	−−−−−−	0	−0.69	−0.6
84620	ST6GAL2	−8.32	0	0.75	−−?−−−	0	−0.67	−0.55
56261	GPCPD1	−8.31	0	0.72	−−?−−−	0	−0.69	−0.53
79930	DOK3	8.3	0	1.27	++?+++	0	0.6	0.47
3754	KCNF1	−8.3	0	0.78	−−−−−−	0	−0.74	−0.61
4792	NFKBIA	8.28	2.22E-16	1.54	++?+++	5.22E-12	0.74	0.58
29906	ST8SIA5	−8.26	2.22E-16	0.79	−−?−−−	5.22E-12	−0.63	−0.49
26471	NUPR1	8.19	2.22E-16	1.66	++++++	5.22E-12	0.71	0.56
1802	DPH2	−8.15	4.44E-16	0.89	−−?−−−	1.04E-11		−0.52
887	CCKBR	−8.13	4.44E-16	0.71	−−−−−−	1.04E-11	−0.72	−0.61
26524	LATS2	8.12	4.44E-16	1.57	++?+++	1.04E-11	0.71	0.57
3017	HIST1H2BD	8.11	4.44E-16	1.46	++++++	1.04E-11		0.5

^$^Average fold change.

^*^data from Zhang *et al.* study^11^.

^#^“+/−/?” indicates up/down and missing.
