# Supplementary material for: Integrated genomic approaches identify major pathways and upstream regulators in late onset Alzheimer’s disease
Source: Sci Rep. 2015 Jul 23;5:12393. doi: 10.1038/srep12393 (PMC4511863; doi:10.1038/srep12393)
Supplement: Supplementary Information [file srep12393-s1.pdf]

Integrated genomic approaches identify major pathways and upstream regulators in late onset Alzheimer's disease

Xinzhong Li, Robert Belshaw, Jintao Long, Taigang He, James Scott

Supplementary Information content lists:

Supplementary Figure S1. Scatter plot between two correlation coefficients: gene expression and Braak stagin vs. gene expression and frontal atrophy.

Supplementary Figure S2. Complement System pathway in late onset Alzheimer's disease.

Supplementary Figure S3. Mechanistic network for upstream regulator LPS, STAT3, TLR4, and NFKBIA predicted by IPA for up-regulated DEGs.

Supplementary Figure S4. Gene set enrichment analysis (GSEA) for NFKB. GSEA identified the NFKB gene set containing up-regulated LOAD DGEs as enriched in old people.

Supplementary Figure S5. Protein-protein interaction network for the top most significantly expressed genes in LOAD and their first neighbours.

Supplementary Figure S6: Protein-protein interaction network for the 23 known LOAD GWAS genes and their first neighbours.

Supplementary Figure S7. Normal Q-Q plot for the meta-analysis.

Supplementary Table S1. Number of samples and genes included in our meta-analysis.

Supplementary Table S2. The full list of 3124 DEGs in an Excel file.

Supplementary Table S3. Top co-expression modules with enriched DEGs.

Supplementary Table S4. The top 30 DEGs mapped to six different brain regions in AD.

Supplementary Table S5. GWAS genes in our meta-analysis plus the 23 known GWAS genes in AD, seven of which were identified as DEGs.

Supplementary Table S6. IPA canonical pathway analysis for LOAD including significant pathways identified by up-regulated and down-regulated DEGs.

Supplementary Table S7. IPA canonical pathway analysis including up-regulated and down-regulated DEGs in ageing.

Supplementary Table S8. Gene set enrichment analysis (GSEA) for ageing with KEGG pathways and four gene sets of DEGs in LOAD.

Supplementary Table S9. IPA upstream regulator analysis for up- and down-regulated DEGs in LOAD

Supplementary Table S10. List of 36 target genes of RICTOR in LOAD.

Supplementary Table S11. IPA upstream regulator analysis for up- and down-regulated ageing.

Supplementary Table S12. Number of neighbours for the top DEGs in the PPI DEG network.

Supplementary Table S13. Number of neighbours for GWAS genes in PPI GWAS network.

Material and methods: Detail of data collection and pre-processing.

Supplementary Figure S1. **Scatter plot between two correlation coefficients: gene expression and Braak stage vs. gene expression and frontal atrophy**

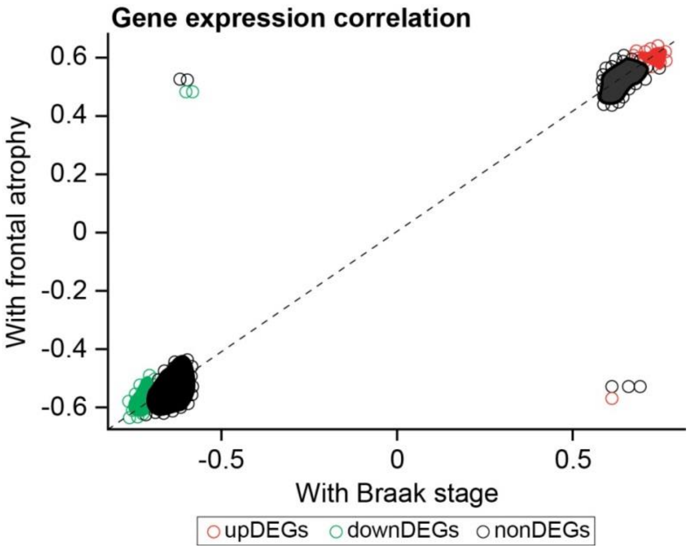

Supplementary Figure S1. Scatter plot between two correlation coefficients: gene expression and Braak stage vs. gene expression and frontal atrophy. Data came from Zhang B. et al's study [1] (data set GSE4470). Genes without correlation to Braak and frontal atrophy were discarded in this figure. Up regulated DEGs are highlighted by red points and down regulated DEGs by green points. DEGs tend to have higher correlation with Braak or frontal atrophy compared to non-DEGs (pval<2.2E-16, t-test). Eight outliers have been removed for enrichment analysis in the main text.

Supplementary Figure S2. Complement System pathway in late onset Alzheimer's disease

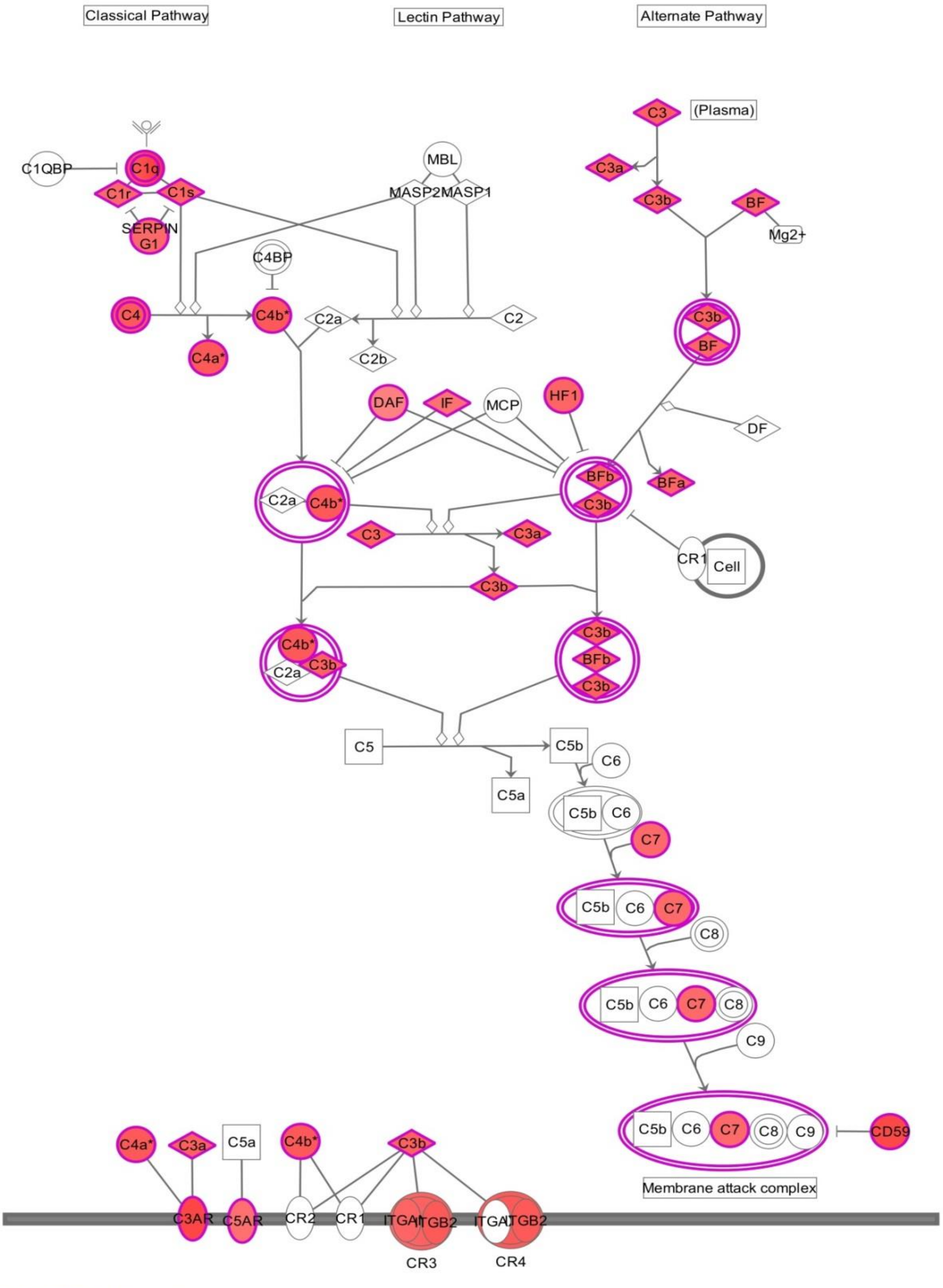

© 2000-2014 QIAGEN. All rights reserved.

Supplementary Figure S2. Complement System pathway in late onset Alzheimer's disease. It is among the top 10 pathways identified by IP for the up regulated DEGs. However no down regulated DEGs founded. 16 (red highlighted) out of in total 33 molecules are up regulated DEGs. See Figure 3 and Supplementary Table S2 online.

Supplementary Figure S3. Mechanistic network for upstream regulators LPS, STAT3, TLR4 and NFKBIA predicted by IPA for upregulated DEGs.

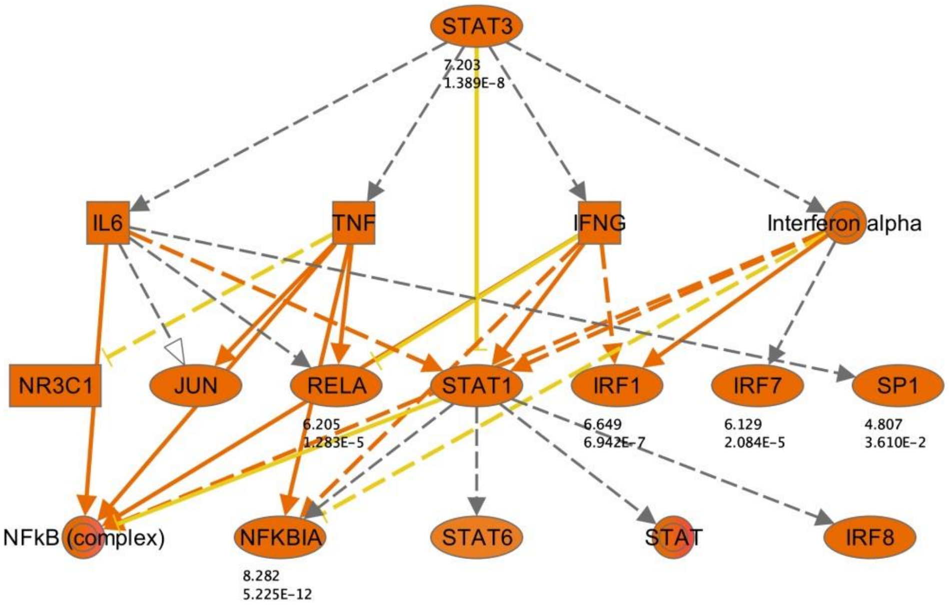

© 2000-2014 QIAGEN. All rights reserved.

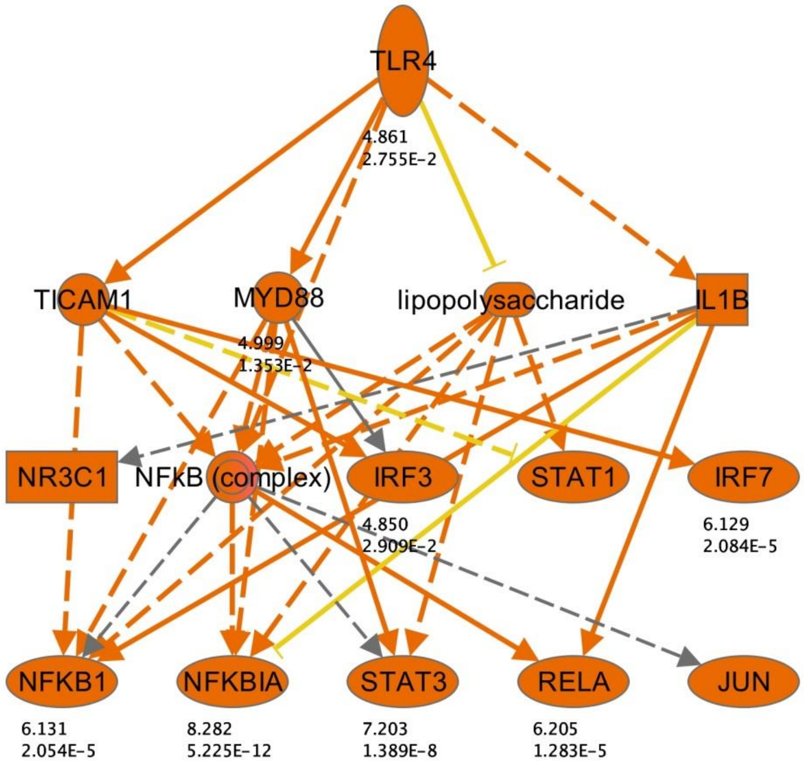

© 2000-2014 QIAGEN. All rights reserved.

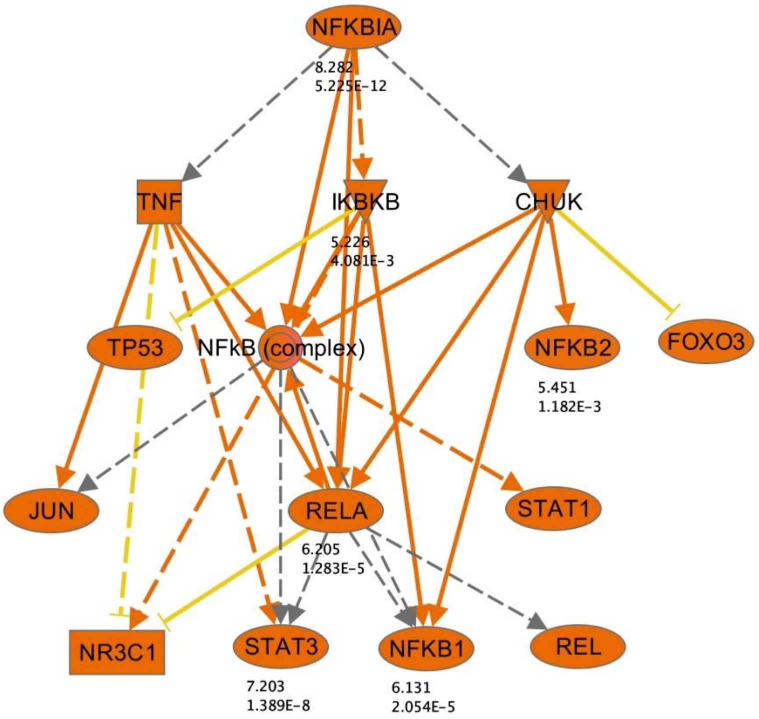

© 2000-2014 QIAGEN. All rights reserved.

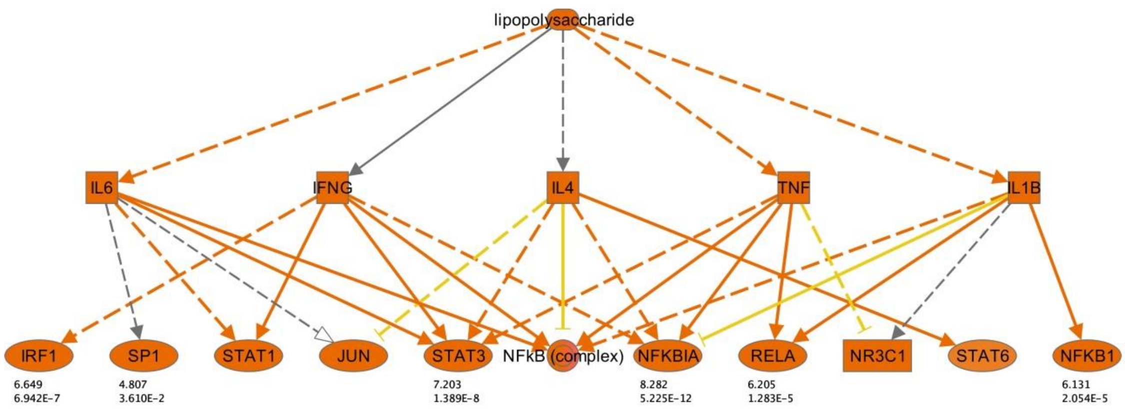

© 2000-2014 QIAGEN. All rights reserved.

Supplementary Figure S3. Mechanistic network for upstream regulators LPS, STAT3, TLR4, and NFKBIA predicted by IPA for upregulated DEGs. DEGs are indicated by their metaZscores and Bonferroni adjusted Pvalues (see Supplementary Table S2 for data detail). The NFKB complex is a key node in these networks.

Supplementary Fig. S4. Gene set enrichment analysis (GSEA) for NFKB.

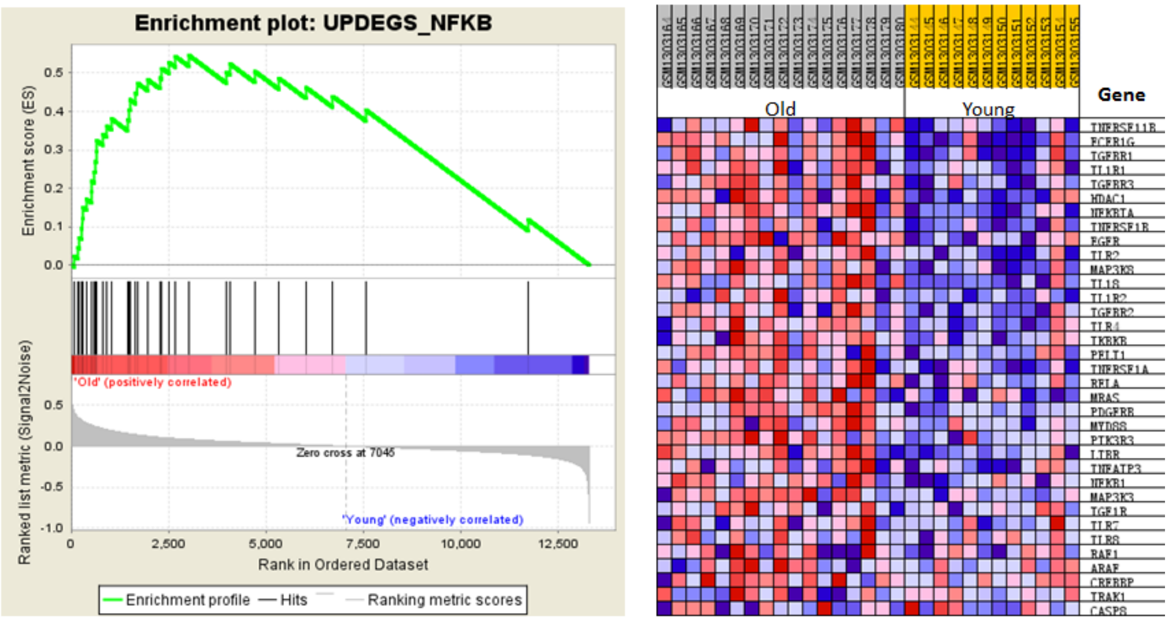

Supplementary Figure S4. Gene set enrichment analysis (GSEA) for NFKB. GSEA confirms the NFKB geneset containing up regulated LOAD DGEs is enriched in old people. Microarray data for ageing (GSE53890) were analysed using GSEA software to identify significant gene sets. The enrichment plot on the left shows the distribution of genes in the set that are correlated with the old or young phenotype. The heatmap on the right shows where the gene expression is relatively high (red) or low (blue) for each gene in the indicated sample.

Supplementary Figure S5: **Protein-protein interaction network for the top 30 most significantly expressed genes in LOAD and their first neighbours.**

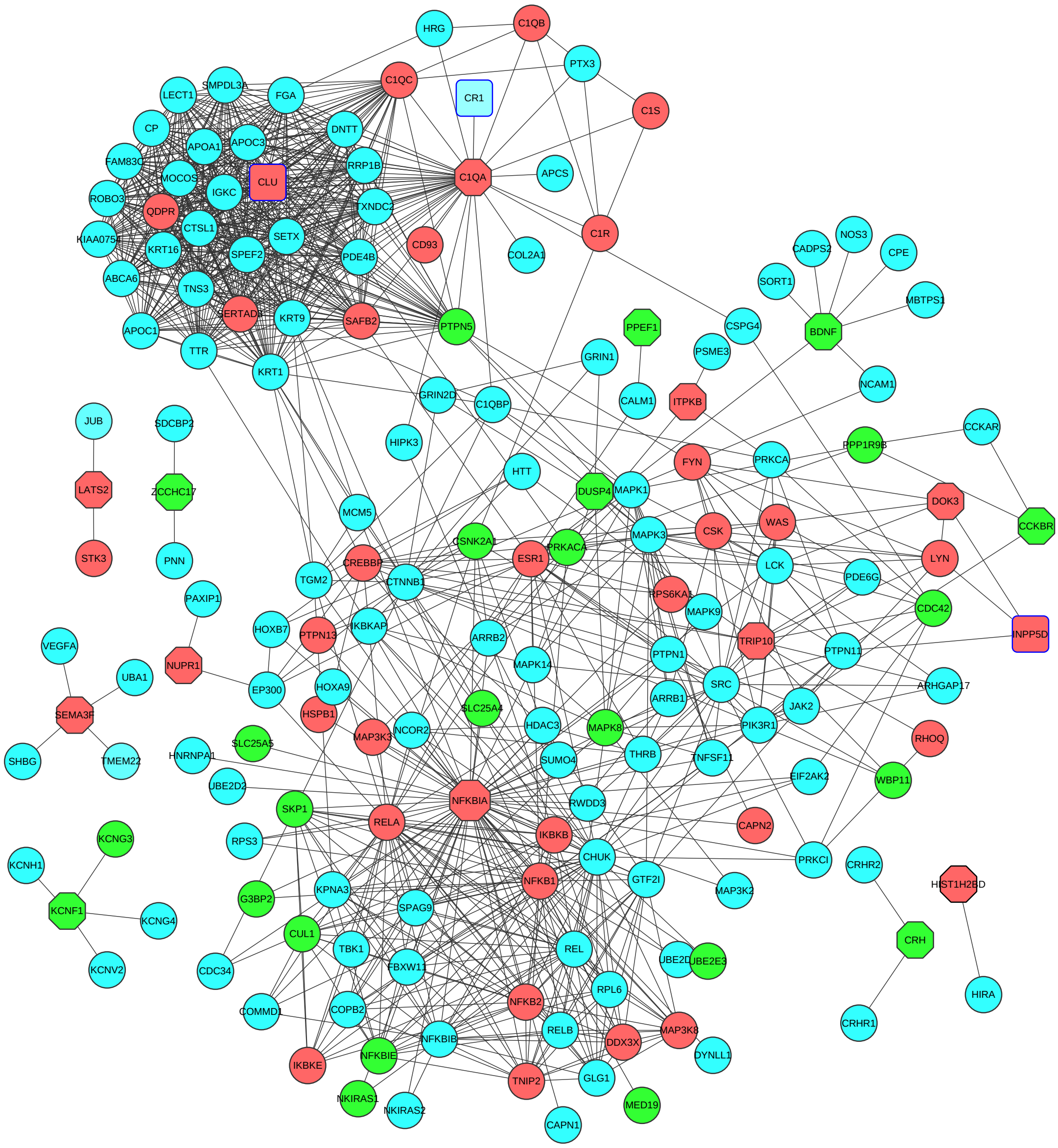

Supplementary Figure S5. Protein-protein interaction network for the top 30 most significantly expressed genes in LOAD and their first neighbours. Among the top 30 DEGs, 16 (octagons, red/green indicates up/down regulation) mapped to the human PPI and their first neighbour. This first neighbour network (FBN) contains 175 nodes and 998 edges. Three GWAS genes are included (squares): INPP5D and CLU are up-regulated DEGs, CR1 is not a DEG. CLU is linked to 32 genes including five DEGs: C1QA, C1QC, QDPR, SERTAD3 and SAFB2. Both CLU and C1QA are ageing relevant genes. NFKBIA, up-regulated, connects to 64 genes in this sub-network including 13 up-regulated DEGs and 10 down-regulated DEGs. For other hubs see Supplementary Table S10.

Supplementary Figure S6: Protein-protein interaction network for the 23 known LOAD GWAS genes and their first neighbours

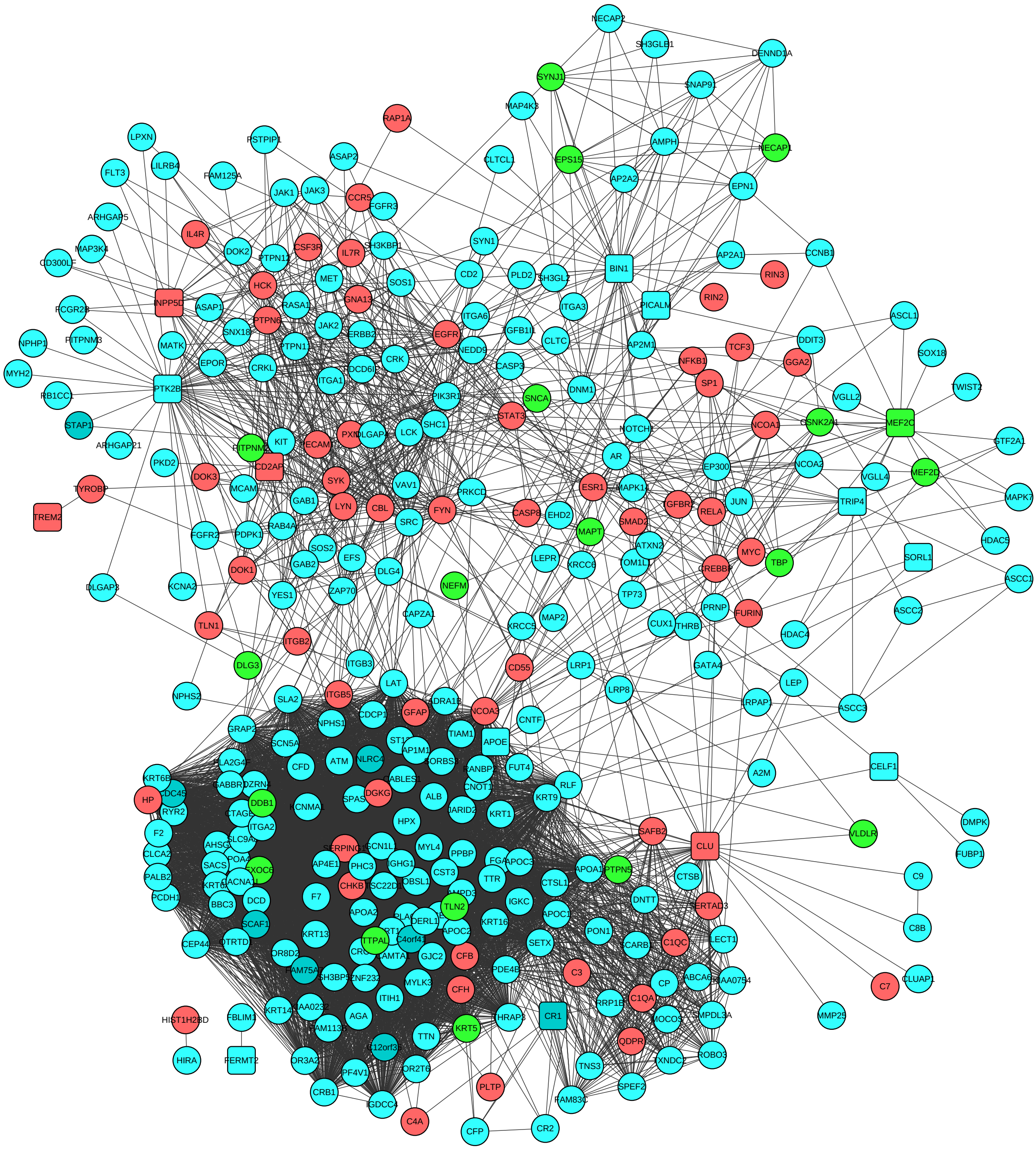

Supplementary Figure S6: Protein-protein interaction network for the 23 known LOAD GWAS genes and their first neighbours. 14 out of 23 GWAS hits (square shaped) were mapped to PPI, their FBN contains 332 nodes and 7692 edges with average 23 neighbours per node higher than the ratio of connections in whole human PPI (OR=5.29, 95%CI 4.64~5.82, pvalue < 2.2e-16). 78 nodes are DEGs (59 up and 19 down, OR=1.45 with 95%CI 1.11~1.89, pvalue=6.82E-3) including 2 top 30DEGs. TYROBP is the 8th most significant DEGs in this sub-network, links to SYK, TREM2 and ZAP70. APOE is the most top hub (124 FBs), PTK2B is the second top hub with 55 FBs. See Supplementary Table S11

Supplementary Figure S7. Normal Q-Q plot for the meta-analysis

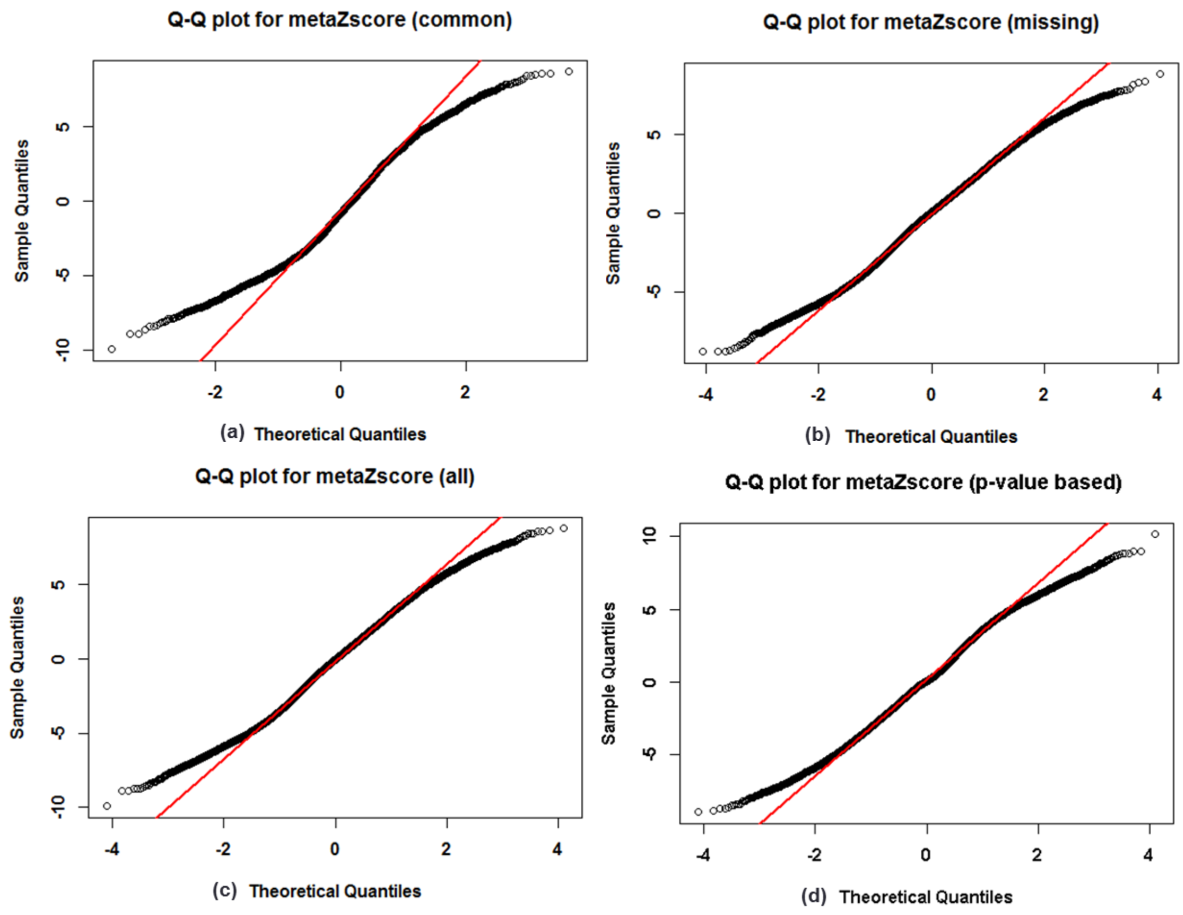

Supplementary Figure S7. Normal Q-Q plot for the meta-analysis. Q-Q plot of the metaZscore calculated for those 3838 common genes across six studies (a); and those missing in at least one study (b); all 23530 genes (c). The distributions are approximated to normal distribution with two fat tails. Clearly the normality of the overall metaZscore is improved by merging data from those incompleted genes (missed in at least one study) to common genes. metaZscore of sample-size weighted P-value combined meta-analysis has similar normality (d).

Supplementary Table S1. Number of samples and genes included in our meta-analysis.

Platform	Study	EntrezGene	Case	Control	Sum
GPL570	GSE5281	13424	23	9	32
GPL570	GSE48350	14903	21	23	44
GPL2700	GSE15222	3995	30	37	67
GPL4372	GSE33000	21576	249	88	337
GPL4372	GSE44770	21576	116	39	155
GPL6244	GSE36980	18922	11	16	27
Total	6	23530	450	212	662

Supplementary Table S2. The full list of 3124 DEGs in an excel file.

Supplementary Table S3. Top co-expression modules with enriched DEGs. Zhang et al. used 1,647 post mortem brain tissues from LOAD patients and non-demented subjects to build the gene co-expression regulatory network, and 20 top modules were identified. Fisher test revealed that half of these modules were enriched with DEGs identified in our meta-analysis.

Module	Rank	Function	EntrezGene*	DEGs	ratio	fisher.pvalue	OR
Yellow	1	immune and microglia	952	444	0.47	4.93E-146	6.49
Pink	2	glutathione transferase	529	78	0.15	1.73E-01	1.13
Gray 1	3	cell junction	43	1	0.02	9.98E-01	0.16
Seashell	4	coated vesicle	229	91	0.4	1.09E-23	4.41
Red 3	5	ribosome	46	3	0.07	9.54E-01	0.46
Green yellow	6	unfolded protein	569	145	0.25	2.06E-15	2.29
Red	7	nerve myelination and oligodendrocytes	786	86	0.11	9.80E-01	0.8
Gold 2	8	axon growth repulsion	67	12	0.18	1.72E-01	1.43
Tan	9	extracellular matrix and choroid plexus cells	597	137	0.23	4.81E-11	1.99
Gold 3	10	dynein complex	65	6	0.09	8.78E-01	0.66
Light yellow	11	mRNA cleavage	276	43	0.16	1.48E-01	1.21
Brown 2	12	olfactory perception	53	3	0.06	9.78E-01	0.39
Dark cyan	13	steroid biosynthesis	84	34	0.4	5.29E-10	4.48
Khaki	14	GABA biosynthesis and astrocytes	229	28	0.12	7.10E-01	0.91
Grey 60	15	Ser/Thr kinase receptor	401	125	0.31	5.71E-21	3.04
Purple	16	synaptic transmission and neurons	665	224	0.34	8.89E-43	3.5
Green 4	17	cell cycle	54	0	0	1.00E+00	0
Honey dew	18	muscle contraction	121	33	0.27	3.23E-05	2.46
Red 2	19	zinc homeostasis	73	47	0.64	5.45E-24	11.97
Beige	20	glucose homeostasis	73	16	0.22	2.81E-02	1.84

*genes without EntrezGene ID were excluded.

Supplementary Table S4. The top 30 DEGs mapped to six different brain regions in AD. GSE5281 contains six brain regions: hippocampus (HIP); entorhinal cortex (EC); medial temporal gyrus (MTG); posterior cingulate (PC); primary visual cortex (PVC), and superior frontal gyrus (SFG). DEGs in each region were identified by limma and then the top meta-analysis DEGs were mapped. C1QA did not present in GSE5281.

GSE5281			HIP			EC			MTG	
EntrezGene	Symbol	logFC	P.Value	adj.P.Val	logFC	P.Value	adj.P.Val	logFC	P.Value	adj.P.Val
63974	NEUROD6	-0.57	2.51E-01	3.96E-01	-0.83	9.12E-05	7.11E-04	-1.24	3.60E-05	3.08E-04
51.538	ZCCHC17	-0.93	6.62E-05	1.09E-03	-0.94	3.00E-05	2.94E-04	-1.75	3.99E-09	8.24E-07
5475	PPEF1	-0.05	7.80E-01	8.58E-01	-1.16	5.43E-03	1.90E-02	-1.07	4.31E-04	2.18E-03
63905	MANBAL	-1.25	1.07E-06	6.23E-05	-1.74	6.10E-11	3.69E-08	-0.87	4.06E-04	2.08E-03
627	BDNF	-1.28	1.54E-02	5.25E-02	-0.38	3.71E-01	5.07E-01	-1.31	1.00E-05	1.14E-04
1392	CRH	-0.65	1.06E-01	2.16E-01	-0.04	9.12E-01	9.46E-01	-0.73	9.98E-05	6.85E-04
3707	ITPKB	1.23	2.02E-02	6.39E-02	3.45	4.34E-09	4.26E-07	3.07	4.92E-09	9.07E-07
388341	FAM211A	-0.86	3.79E-04	3.65E-03	-0.45	2.52E-03	1.02E-02	-0.25	1.91E-01	2.90E-01
2289	FKBP5	-0.66	9.88E-03	3.80E-02	1.37	6.51E-03	2.20E-02	1.07	1.14E-02	3.04E-02
64231	MS4A6A	0.46	5.27E-02	1.29E-01	1.02	2.66E-03	1.07E-02	0.67	3.63E-02	7.75E-02
78991	PCYOX1L	-1.22	4.10E-07	3.28E-05	-1.31	5.07E-06	7.52E-05	-1.98	3.07E-08	2.32E-06
1846	DUSP4	-1.95	5.22E-04	4.56E-03	0.05	7.04E-01	7.98E-01	-0.52	3.23E-02	7.04E-02
7108	TM7SF2	-0.74	1.94E-03	1.15E-02	-1.14	3.29E-08	1.77E-06	-1.23	5.95E-06	7.73E-05
6405	SEMA3F	0.95	3.41E-03	1.74E-02	0.57	1.51E-01	2.59E-01	1.87	6.89E-07	1.67E-05
9322	TRIP10	0.47	1.72E-03	1.06E-02	0.49	1.90E-02	5.18E-02	0.74	1.59E-05	1.64E-04
10184	LHFPL2	-0.47	6.52E-02	1.51E-01	0.74	5.08E-03	1.80E-02	0.12	6.14E-01	7.10E-01
9315	NREP	-0.72	1.81E-03	1.10E-02	0.47	1.07E-01	2.00E-01	-1.55	9.08E-10	4.38E-07
381	ARF5	-1.06	5.42E-06	1.90E-04	-0.10	6.15E-01	7.27E-01	-0.49	1.60E-02	4.00E-02
84620	ST6GAL2	-0.61	4.41E-02	1.13E-01	-2.06	7.53E-10	1.35E-07	-0.41	4.18E-02	8.69E-02
56261	GPCPD1	-0.63	6.90E-04	5.55E-03	0.58	3.53E-04	2.11E-03	-0.77	1.07E-02	2.89E-02
79930	DOK3	0.85	5.89E-04	4.96E-03	-0.40	1.13E-01	2.08E-01	0.52	1.77E-02	4.33E-02
3754	KCNF1	-0.24	1.13E-01	2.26E-01	-1.11	6.80E-05	5.61E-04	-1.06	5.45E-05	4.25E-04
4792	NFKBIA	1.24	2.24E-03	1.27E-02	2.58	6.25E-08	2.76E-06	1.70	1.88E-07	6.82E-06
29906	ST8SIA5	-0.47	5.09E-02	1.26E-01	-0.34	4.44E-02	1.01E-01	-0.76	1.06E-03	4.51E-03
26471	NUPR1	0.24	2.49E-01	3.95E-01	-0.03	8.71E-01	9.19E-01	0.61	9.30E-03	2.58E-02
1802	DPH2	-0.10	4.91E-01	6.33E-01	-1.07	7.68E-06	1.04E-04	-0.21	2.34E-01	3.40E-01
887	CCKBR	-0.50	3.22E-02	9.01E-02	-1.44	4.34E-05	3.94E-04	-1.71	8.61E-07	1.92E-05
26524	LATS2	1.73	9.60E-04	7.01E-03	2.13	9.38E-06	1.21E-04	2.40	2.56E-07	8.46E-06
3017	HIST1H2BD	0.31	2.73E-01	4.21E-01	0.04	7.74E-01	8.50E-01	0.32	1.68E-01	2.62E-01
GSE5281			PC			PVC			SFG	
EntrezGene	Symbol	logFC	P.Value	adj.P.Val	logFC	P.Value	adj.P.Val	logFC	P.Value	adj.P.Val
63974	NEUROD6	-0.53	9.86E-02	1.88E-01	0.11	6.10E-01	7.77E-01	-1.18	4.37E-06	6.74E-04
51538	ZCCHC17	-0.60	3.32E-03	1.58E-02	-0.40	4.79E-02	1.94E-01	-0.62	3.64E-04	7.31E-03
5475	PPEF1	-1.45	1.28E-04	1.79E-03	-0.14	2.50E-01	4.68E-01	-0.78	3.96E-03	3.05E-02
63905	MANBAL	-1.09	4.63E-04	4.20E-03	-0.45	2.92E-02	1.56E-01	-0.28	2.56E-01	4.42E-01
627	BDNF	-1.03	1.03E-03	7.01E-03	-0.59	4.51E-02	1.88E-01	-0.84	3.04E-04	6.66E-03
1392	CRH	-1.16	2.06E-02	5.91E-02	-0.64	5.94E-02	2.14E-01	-0.68	2.14E-02	8.95E-02
3707	ITPKB	1.13	9.35E-04	6.58E-03	1.40	3.88E-04	5.61E-02	2.36	2.22E-07	1.74E-04
388341	FAM211A	-0.51	9.28E-03	3.32E-02	-0.16	3.04E-01	5.20E-01	-0.30	5.01E-02	1.55E-01
2289	FKBP5	-0.56	2.49E-02	6.78E-02	0.61	3.79E-02	1.74E-01	0.74	2.14E-02	8.94E-02
64231	MS4A6A	0.40	8.17E-02	1.64E-01	0.47	1.45E-02	1.20E-01	0.25	3.62E-01	5.52E-01
78991	PCYOX1L	-1.24	9.13E-04	6.49E-03	-1.15	1.31E-04	4.53E-02	-1.20	1.37E-04	4.11E-03
1846	DUSP4	-0.39	4.69E-02	1.08E-01	-0.93	1.89E-03	7.35E-02	-0.42	5.20E-03	3.61E-02
7108	TM7SF2	-0.24	2.19E-01	3.40E-01	-0.02	8.95E-01	9.49E-01	-0.66	5.64E-04	9.38E-03
6405	SEMA3F	1.72	1.28E-07	3.79E-05	0.97	6.21E-03	9.57E-02	0.67	3.63E-02	1.26E-01
9322	TRIP10	0.54	1.11E-02	3.77E-02	0.49	9.74E-03	1.06E-01	0.45	4.60E-03	3.34E-02
10184	LHFPL2	0.16	3.47E-01	4.78E-01	0.45	5.66E-02	2.09E-01	0.55	1.28E-02	6.40E-02
9315	NREP	-0.68	5.61E-04	4.73E-03	-0.66	4.21E-02	1.82E-01	-0.82	5.72E-04	9.46E-03
381	ARF5	-0.20	3.72E-01	5.02E-01	-0.01	9.78E-01	9.89E-01	-0.96	1.09E-06	3.63E-04
84620	ST6GAL2	-1.20	7.16E-04	5.54E-03	-1.20	2.25E-03	7.67E-02	-0.45	4.07E-02	1.36E-01
56261	GPCPD1	-0.61	1.91E-02	5.60E-02	-0.85	4.50E-03	8.94E-02	-0.88	5.55E-04	9.29E-03
79930	DOK3	0.87	2.98E-04	3.10E-03	0.99	9.67E-05	4.49E-02	0.40	4.03E-02	1.35E-01
3754	KCNF1	-0.74	4.51E-03	1.97E-02	-0.19	2.21E-01	4.34E-01	-0.69	1.25E-04	3.89E-03
4792	NFKBIA	0.92	1.42E-02	4.52E-02	0.72	1.22E-02	1.13E-01	1.25	1.06E-04	3.57E-03
29906	ST8SIA5	-0.93	2.68E-04	2.91E-03	-0.37	2.36E-02	1.43E-01	-0.67	1.80E-03	1.88E-02
26471	NUPR1	0.22	2.83E-01	4.12E-01	0.44	3.84E-02	1.75E-01	1.13	3.58E-06	6.22E-04
1802	DPH2	0.19	1.41E-01	2.45E-01	0.19	1.90E-01	3.99E-01	-0.18	2.34E-01	4.18E-01

887	CCKBR	-0.95	2.14E-03	1.17E-02	-0.72	1.56E-03	6.96E-02	-0.72	8.22E-03	4.81E-02
26524	LATS2	1.23	2.55E-03	1.32E-02	1.02	6.36E-03	9.60E-02	1.84	5.05E-06	7.18E-04
3017	HIST1H2BD	0.47	7.21E-02	1.49E-01	0.34	1.49E-01	3.46E-01	0.41	1.11E-01	2.59E-01

Supplementary Table S5. GWAS genes in our meta-analysis. The 23 known GWAS genes in AD, seven of which were identified as DEGs

EntrezGene	Symbol	metaZscore	metaPval	avgFC	Effect#	BonfPval	Braak*	atrophy*
64231	MS4A6A	8.57	0	1.43	++++++	0	0.62	0.51
23607	CD2AP	6.73	1.73E-11	1.15	++?+++	4.08E-07	0.68	0.57
3635	INPP5D	6.64	3.20E-11	1.25	?+?+++	7.52E-07	0.66	0.56
4208	MEF2C	-6.56	5.32E-11	0.73	------	1.25E-06	-0.65	-0.51
1191	CLU	6.23	4.75E-10	1.31	++?++-	1.12E-05	0.62	0.46
54209	TREM2	6.02	1.76E-09	1.26	?+++++	4.13E-05		
10347	ABCA7	5.32	1.05E-07	1.13	++?+++	2.47E-03		
9325	TRIP4	4.27	1.97E-05	1.08	++?++-	4.64E-01	0.64	0.57
23646	PLD3	-3.77	1.60E-04	0.84	--?---	1	-0.64	-0.57
10979	FERMT2	3.35	7.98E-04	1.21	+-+++-	1	0.6	0.49
51314	TXNDC3	3.2	1.38E-03	1.10	???+++	1		
6653	SORL1	-2.97	2.93E-03	0.89	-++---	1		
1378	CR1	2.63	8.52E-03	1.03	-+?+++	1		
10658	CELF1	1.86	6.23E-02	0.95	--?+++	1		0.48
123041	SLC24A4	1.77	7.68E-02	1.11	++?+++	1		
2041	EPHA1	1.35	1.76E-01	1.01	???++-	1		
55063	ZCWPW1	1.31	1.91E-01	1.01	-+?++-	1		
8301	PICALM	-1.22	2.23E-01	0.95	-+?--+	1		
348	APOE	1.13	2.57E-01	1.12	+-?-++	1		
57091	CASS4	-0.38	7.01E-01	1.02	?+?--+	1		
2185	PTK2B	0.38	7.06E-01	0.99	--?+++	1		-0.41
274	BIN1	-0.31	7.55E-01	0.96	--?-++	1		
3127	HLA_DRB5	0.32	4.29E-01	1.43	???++-	1		

*data from Zhang et.al [1] ; # “+/-/?” indicates up/down and missing

Supplementary Table S6. IPA canonical pathway analysis for LOAD including significant pathways identified by upregulated and downregulated DEGs.

Supplementary Table S4.					
Ingenuity Canonical Pathways	DEGs	Genes In Pathway	Ratio	z.score	adjPval
Pathways from up-regulated DEGs					
Production of Nitric Oxide and Reactive Oxygen Species in Macrophages	44	180	0.244	4.938	1.26E-12
NF-κB Signalling	41	173	0.237	5.154	1.26E-11
Role of Macrophages, Fibroblasts and Endothelial Cells in Rheumatoid Arthritis	56	298	0.188		1.26E-11
LXR/RXR Activation	33	121	0.273	-3.182	3.16E-11
IL-8 Signalling	40	183	0.219	6	2.09E-10

B Cell Receptor Signalling	39	176	0.222		2.09E-10
IL-10 Signalling	23	69	0.333		7.76E-10
IL-6 Signalling	30	116	0.259	5.385	8.32E-10
Complement System	16	33	0.485	0.832	1.02E-09
Acute Phase Response Signalling	36	169	0.213	4.747	2.82E-09
Dendritic Cell Maturation	37	179	0.207		3.55E-09
Hepatic Fibrosis / Hepatic Stellate Cell Activation	38	197	0.193		1.51E-08
Role of Pattern Recognition Receptors in Recognition of Bacteria and Viruses	29	127	0.228	4.69	2.82E-08
Molecular Mechanisms of Cancer	55	364	0.151		3.47E-08
PPAR Signalling	24	94	0.255	-3.266	6.31E-08
Toll-like Receptor Signalling	21	74	0.284	3.357	6.92E-08
Tec Kinase Signalling	32	158	0.203	5.196	6.92E-08
Type I Diabetes Mellitus Signalling	26	110	0.236	4.243	6.92E-08
TREM1 Signalling	21	75	0.28	4.583	8.32E-08
PI3K Signalling in B Lymphocytes	28	128	0.219	4.707	1.00E-07
Death Receptor Signalling	23	92	0.25	2.711	1.51E-07
Role of Osteoblasts, Osteoclasts and Chondrocytes in Rheumatoid Arthritis	38	218	0.174		1.82E-07
p38 MAPK Signalling	26	117	0.222	4.082	2.14E-07
Role of NFAT in Regulation of the Immune Response	32	171	0.187	4.583	3.72E-07
Leukocyte Extravasation Signalling	35	198	0.177	2.858	3.72E-07
iNOS Signalling	15	44	0.341	3.873	5.37E-07
Atherosclerosis Signalling	26	123	0.211		5.37E-07
Acute Myeloid Leukemia Signalling	20	77	0.26	3.3	5.37E-07
T Helper Cell Differentiation	19	71	0.268		6.76E-07
CD28 Signalling in T Helper Cells	25	118	0.212	3.3	8.71E-07
Hepatic Cholestasis	30	162	0.185		1.05E-06
Crosstalk between Dendritic Cells and Natural Killer Cells	21	89	0.236		1.32E-06
Signalling by Rho Family GTPases	37	234	0.158	5.568	2.29E-06
Fcγ Receptor-mediated Phagocytosis in Macrophages and Monocytes	21	93	0.226	4.146	2.63E-06
Chronic Myeloid Leukemia Signalling	21	93	0.226		2.63E-06
PPARα/RXRα Activation	31	179	0.173	-4.025	2.63E-06
IL-12 Signalling and Production in Macrophages	26	135	0.193		2.75E-06
PKCθ Signalling in T Lymphocytes	24	118	0.203	4.899	2.75E-06
PTEN Signalling	24	118	0.203	-3.411	2.75E-06
HIPPO Signalling	20	86	0.233	0	2.75E-06
Regulation of IL-2 Expression in Activated and Anergic T Lymphocytes	19	79	0.241		3.02E-06
HMGB1 Signalling	24	120	0.2	4.796	3.55E-06
Apoptosis Signalling	20	89	0.225	-0.447	4.57E-06
TWEAK Signalling	12	34	0.353	2.309	5.13E-06
Granulocyte Adhesion and Diapedesis	30	178	0.169		5.37E-06
IL-4 Signalling	18	76	0.237		6.76E-06
fMLP Signalling in Neutrophils	22	108	0.204	4.025	6.76E-06
IL-17A Signalling in Fibroblasts	12	35	0.343		6.76E-06
Phospholipase C Signalling	36	238	0.151	5.385	7.41E-06
Gαq Signalling	26	147	0.177	4.379	1.17E-05
Altered T Cell and B Cell Signalling in Rheumatoid Arthritis	19	88	0.216		1.41E-05
RANK Signalling in Osteoclasts	19	88	0.216		1.41E-05
Colorectal Cancer Metastasis Signalling	35	236	0.148	5.292	1.41E-05
ERK/MAPK Signalling	30	188	0.16	3.651	1.45E-05
HGF Signalling	21	105	0.2	3.771	1.45E-05
LPS-stimulated MAPK Signalling	17	73	0.233	3.5	1.51E-05
Glucocorticoid Receptor Signalling	37	261	0.142		2.00E-05
Estrogen-Dependent Breast Cancer Signalling	15	62	0.242	3.606	3.55E-05
Germ Cell-Sertoli Cell Junction Signalling	26	160	0.163		5.13E-05
PI3K/AKT Signalling	22	123	0.179	1.342	5.25E-05
Glioma Invasiveness Signalling	14	57	0.246	2.673	5.75E-05
Pancreatic Adenocarcinoma Signalling	20	106	0.189	3.207	5.75E-05
NF-κB Activation by Viruses	16	73	0.219	3.873	6.17E-05
STAT3 Pathway	16	73	0.219		6.17E-05
NGF Signalling	20	107	0.187	4.472	6.31E-05
Antigen Presentation Pathway	11	37	0.297		6.92E-05
iCOS-iCOSL Signalling in T Helper Cells	20	108	0.185	2.496	7.08E-05
IL-1 Signalling	18	91	0.198	3.742	7.41E-05
Erythropoietin Signalling	15	67	0.224		8.13E-05

RAR Activation	27	176	0.153		8.51E-05
Role of RIG1-like Receptors in Antiviral Innate Immunity	12	45	0.267	3.162	8.91E-05
Induction of Apoptosis by HIV1	14	60	0.233	2.673	9.12E-05
Macropinocytosis Signalling	15	68	0.221	3	9.33E-05
Axonal Guidance Signalling	50	435	0.115		1.23E-04
Role of IL-17A in Arthritis	13	54	0.241		1.23E-04
Rac Signalling	19	104	0.183	4.243	1.26E-04
Thrombin Signalling	28	190	0.147	5	1.29E-04
Role of PKR in Interferon Induction and Antiviral Response	11	40	0.275		1.35E-04
Cardiac Hypertrophy Signalling	31	223	0.139	4.811	1.35E-04
RhoGDI Signalling	26	173	0.15	-3.71	1.55E-04
T Cell Receptor Signalling	18	97	0.186		1.55E-04
Regulation of the Epithelial-Mesenchymal Transition Pathway	27	184	0.147		1.66E-04
IL-17 Signalling	15	72	0.208		1.66E-04
JAK/Stat Signalling	15	72	0.208	1.807	1.66E-04
TNFR1 Signalling	12	49	0.245	2.714	1.91E-04
Glioblastoma Multiforme Signalling	23	146	0.158	3.545	1.95E-04
Sphingosine-1-phosphate Signalling	19	109	0.174	3.742	2.19E-04
TNFR2 Signalling	9	29	0.31	2.121	2.29E-04
IL-15 Signalling	14	66	0.212		2.34E-04
Role of Tissue Factor in Cancer	19	110	0.173		2.40E-04
Interferon Signalling	10	36	0.278	1.414	2.45E-04
Aryl Hydrocarbon Receptor Signalling	22	140	0.157	3.153	2.75E-04
Role of IL-17F in Allergic Inflammatory Airway Diseases	11	44	0.25		2.95E-04
Integrin Signalling	28	201	0.139	4.811	2.95E-04
CD27 Signalling in Lymphocytes	12	52	0.231		3.16E-04
Role of JAK1, JAK2 and TYK2 in Interferon Signalling	8	24	0.333		3.16E-04
Mouse Embryonic Stem Cell Pluripotency	17	95	0.179	3.638	3.39E-04
Protein Kinase A Signalling	44	383	0.115		3.39E-04
April Mediated Signalling	10	38	0.263	2.53	3.72E-04
Ephrin Receptor Signalling	25	174	0.144		3.72E-04
Human Embryonic Stem Cell Pluripotency	21	134	0.157		3.80E-04
Lymphotoxin β Receptor Signalling	12	54	0.222	2.53	4.37E-04
PEDF Signalling	14	71	0.197	3.051	4.68E-04
14-3-3-mediated Signalling	19	117	0.162		4.90E-04
Caveolar-mediated Endocytosis Signalling	14	72	0.194		5.37E-04
Antiproliferative Role of TOB in T Cell Signalling	8	26	0.308		5.50E-04
B Cell Activating Factor Signalling	10	40	0.25	3	5.50E-04
IL-17A Signalling in Airway Cells	13	64	0.203		5.75E-04
Prolactin Signalling	14	73	0.192	3.464	6.03E-04
Prostate Cancer Signalling	15	82	0.183		6.17E-04
Factors Promoting Cardiogenesis in Vertebrates	16	92	0.174		6.92E-04
Oncostatin M Signalling	9	34	0.265	3	7.08E-04
Angiopoietin Signalling	13	66	0.197	0.905	7.59E-04
ILK Signalling	25	187	0.134	4.379	9.55E-04
TGF-β Signalling	15	87	0.172	0.832	1.15E-03
GM-CSF Signalling	12	62	0.194	3.464	1.51E-03
IL-3 Signalling	13	71	0.183	1.941	1.55E-03
Telomerase Signalling	16	99	0.162	2.309	1.55E-03
P2Y Purigenic Receptor Signalling Pathway	18	119	0.151	3.5	1.58E-03
Natural Killer Cell Signalling	17	110	0.155		1.74E-03
Actin Cytoskeleton Signalling	27	218	0.124		1.82E-03
LPS/IL-1 Mediated Inhibition of RXR Function	27	218	0.124	1.147	1.95E-03
Actin Nucleation by ARP-WASP Complex	11	56	0.196	3.317	2.19E-03
Wnt/Ca+ pathway	11	56	0.196	3.317	2.19E-03
CD40 Signalling	12	65	0.185	2.714	2.24E-03
Nur77 Signalling in T Lymphocytes	11	57	0.193		2.45E-03
ErbB2-ErbB3 Signalling	11	57	0.193		2.45E-03
MIF-mediated Glucocorticoid Regulation	8	33	0.242	2.828	2.69E-03
Xenobiotic Metabolism Signalling	31	270	0.115		2.75E-03
HER-2 Signalling in Breast Cancer	13	76	0.171		2.75E-03
Role of IL-17A in Psoriasis	5	13	0.385		2.75E-03
PDGF Signalling	13	77	0.169	3.606	3.09E-03
FAK Signalling	14	87	0.161		3.16E-03
Type II Diabetes Mellitus Signalling	17	117	0.145		3.16E-03
IL-9 Signalling	8	34	0.235	2.121	3.16E-03
B Cell Development	8	34	0.235		3.16E-03
VDR/RXR Activation	13	78	0.167	-1.897	3.39E-03

Neuregulin Signalling	14	88	0.159	3.464	3.47E-03
IL-15 Production	7	27	0.259		3.47E-03
p53 Signalling	15	98	0.153	0	3.47E-03
p70S6K Signalling	17	119	0.143	3.153	3.72E-03
CXCR4 Signalling	20	152	0.132	4.243	3.98E-03
Ceramide Signalling	13	80	0.163	2.496	4.07E-03
Small Cell Lung Cancer Signalling	12	71	0.169		4.37E-03
Cholecystokinin/Gastrin-mediated Signalling	15	101	0.149	3.873	4.57E-03
Communication between Innate and Adaptive Immune Cells	14	91	0.154		4.57E-03
Paxillin Signalling	15	102	0.147	2.121	5.01E-03
Agranulocyte Adhesion and Diapedesis	23	189	0.122		5.01E-03
Sertoli Cell-Sertoli Cell Junction Signalling	22	177	0.124		5.13E-03
Epithelial Adherens Junction Signalling	19	146	0.13		5.62E-03
Calcium-induced T Lymphocyte Apoptosis	11	64	0.172	2.828	5.62E-03
MSP-RON Signalling Pathway	9	46	0.196		5.62E-03
Breast Cancer Regulation by Stathmin1	23	192	0.12		5.62E-03
FLT3 Signalling in Hematopoietic Progenitor Cells	12	74	0.162		5.89E-03
TR/RXR Activation	13	85	0.153		6.61E-03
Extrinsic Prothrombin Activation Pathway	5	16	0.312	0	6.61E-03
4-1BB Signalling in T Lymphocytes	7	31	0.226	2.449	7.24E-03
Graft-versus-Host Disease Signalling	9	48	0.188		7.41E-03
Antioxidant Action of Vitamin C	14	97	0.144	-3.464	7.76E-03
GNRH Signalling	17	129	0.132	2.668	7.76E-03
MIF Regulation of Innate Immunity	8	41	0.195	2.828	9.55E-03
Virus Entry via Endocytic Pathways	13	89	0.146		9.55E-03
OX40 Signalling Pathway	13	89	0.146	1.342	9.55E-03
Growth Hormone Signalling	11	69	0.159	2.53	9.55E-03
Reelin Signalling in Neurons	12	79	0.152		9.55E-03
Pathways from down-regulated DEGs					
Ingenuity Canonical Pathways	DEGs	Genes In Pathway	Ratio	z.score	adjPval
Mitochondrial Dysfunction	37	172	0.215		2.24E-06
Oxidative Phosphorylation	24	110	0.218		4.27E-04
Aspartate Degradation II	5	7	0.714		7.24E-03

Supplementary Table S7. IPA canonical pathway analysis including upregulated and downregulated DEGs in ageing.

**Supplementary table S5. IPA for ageing**					
Ingenuity Canonical Pathways	DEGs	Genes In Pathway	Ratio	z.score	adjPval
Pathways for up regulated DEGs					
EIF2 Signalling	41	169	0.243	5.196	1.05E-10
Germ Cell-Sertoli Cell Junction Signalling	30	154	0.195		1.58E-05
mTOR Signalling	33	181	0.182		1.58E-05
Semaphorin Signalling in Neurons	16	52	0.308		1.62E-05
Integrin Signalling	34	194	0.175	5.303	1.66E-05
Signalling by Rho Family GTPases	37	231	0.16	5.112	4.17E-05
STAT3 Pathway	18	73	0.247		6.17E-05
RhoA Signalling	23	118	0.195	3.71	1.55E-04
RhoGDI Signalling	29	172	0.169	-4.379	1.62E-04
Adipogenesis pathway	23	120	0.192		1.66E-04
ILK Signalling	29	181	0.16	4.536	3.72E-04
Molecular Mechanisms of Cancer	46	359	0.128		5.01E-04
Leukocyte Extravasation Signalling	29	191	0.152	2.683	8.91E-04
Actin Nucleation by ARP-WASP Complex	13	56	0.232	3.606	1.95E-03
Fatty Acid ??-oxidation I	9	29	0.31		2.24E-03
IL-8 Signalling	27	182	0.148	5.099	2.24E-03
Regulation of Cellular Mechanics by Calpain Protease	12	54	0.222	1.89	4.68E-03
Ephrin Receptor Signalling	25	172	0.145		4.68E-03
Dopamine Degradation	7	20	0.35		4.79E-03
Caveolar-mediated Endocytosis Signalling	14	71	0.197		4.79E-03

Glioma Invasiveness Signalling	12	57	0.211	2.887	6.76E-03
Regulation of eIF4 and p70S6K Signalling	21	140	0.15	1.633	7.41E-03
Tryptophan Degradation X (Mammalian, via Tryptamine)	6	16	0.375		7.41E-03
Putrescine Degradation III	6	16	0.375		7.41E-03
Epithelial Adherens Junction Signalling	21	141	0.149		7.59E-03
Aryl Hydrocarbon Receptor Signalling	20	133	0.15	2.496	8.51E-03
FAK Signalling	15	86	0.174		8.51E-03
Clathrin-mediated Endocytosis Signalling	25	184	0.136		8.51E-03
Ethanol Degradation IV	6	17	0.353		8.91E-03

Pathways for down regulated DEGs

Ingenuity.Canonical.Pathways	DEGs	Genes In pathway	Ratio	z.score	adjPval
Breast Cancer Regulation by Stathmin1	43	188	0.229		1.35E-06
Dopamine-DARPP32 Feedback in cAMP Signalling	38	157	0.242	-2.785	1.35E-06
Oxidative Phosphorylation	28	96	0.292		1.35E-06
Signalling by Rho Family GTPases	48	231	0.208	-5.667	1.82E-06
Huntington's Disease Signalling	47	225	0.209		1.82E-06
Mitochondrial Dysfunction	37	157	0.236		1.82E-06
Protein Ubiquitination Pathway	50	251	0.199		2.40E-06
Role of NFAT in Cardiac Hypertrophy	39	174	0.224		2.40E-06
Rac Signalling	28	103	0.272	-5.099	2.40E-06
Axonal Guidance Signalling	71	420	0.169		3.31E-06
G Beta Gamma Signalling	25	88	0.284		3.98E-06
CDK5 Signalling	26	98	0.265	-2.353	9.12E-06
Ephrin Receptor Signalling	36	172	0.209		2.95E-05
Corticotropin Releasing Hormone Signalling	26	105	0.248		3.39E-05
fMLP Signalling in Neutrophils	26	107	0.243	-4.796	4.17E-05
GNRH Signalling	29	127	0.228	-5.385	4.17E-05
CREB Signalling in Neurons	35	169	0.207	-5	4.17E-05
14-3-3-mediated Signalling	27	114	0.237		4.17E-05
Synaptic Long Term Potentiation	27	116	0.233		5.62E-05
CXCR4 Signalling	32	151	0.212	-4.2	5.89E-05
Renin-Angiotensin Signalling	25	108	0.231	-5	1.32E-04
IL-1 Signalling	22	91	0.242	-3.638	2.14E-04
GABA Receptor Signalling	18	66	0.273		2.19E-04
??-Adrenergic Signalling	21	85	0.247	-2.5	2.19E-04
Cardiac Hypertrophy Signalling	39	218	0.179	-5.145	2.95E-04
RhoGDI Signalling	33	172	0.192	4.899	2.95E-04
CCR5 Signalling in Macrophages	17	62	0.274		3.02E-04
IGF-1 Signalling	22	96	0.229	-2.668	4.17E-04
Myc Mediated Apoptosis Signalling	16	58	0.276		4.47E-04
Synaptic Long Term Depression	27	132	0.205	-2.887	4.47E-04
HIPPO Signalling	20	85	0.235	3.464	5.75E-04
Cholecystokinin/Gastrin-mediated Signalling	22	99	0.222	-4.69	6.03E-04
Androgen Signalling	23	110	0.209	-3	1.07E-03
p70S6K Signalling	24	118	0.203	-1.606	1.17E-03
CCR3 Signalling in Eosinophils	23	111	0.207		1.17E-03
Gap Junction Signalling	28	149	0.188		1.32E-03
Aldosterone Signalling in Epithelial Cells	28	150	0.187		1.45E-03
ErbB Signalling	19	85	0.224		1.48E-03
Neuropathic Pain Signalling In Dorsal Horn Neurons	21	99	0.212	-4.583	1.48E-03
Protein Kinase A Signalling	54	365	0.148		1.48E-03
UVC-Induced MAPK Signalling	12	42	0.286	-3.464	2.19E-03
Nitric Oxide Signalling in the Cardiovascular System	20	95	0.211	-3.441	2.19E-03
Melatonin Signalling	16	68	0.235	-2.5	2.40E-03
P2Y Purigenic Receptor Signalling Pathway	23	118	0.195	-4.025	2.45E-03
Glutamate Receptor Signalling	14	56	0.25	-2.449	2.82E-03
NRF2-mediated Oxidative Stress Response	30	175	0.171	-4	3.24E-03
Cdc42 Signalling	23	121	0.19		3.39E-03
Clathrin-mediated Endocytosis Signalling	31	185	0.168		3.39E-03
Remodeling of Epithelial Adherens Junctions	15	64	0.234	-2.828	3.39E-03
G??q Signalling	26	145	0.179	-3.962	3.55E-03
CD28 Signalling in T Helper Cells	21	107	0.196	-4.146	3.55E-03
Reelin Signalling in Neurons	17	79	0.215		3.89E-03
UVB-Induced MAPK Signalling	13	52	0.25	-3.606	3.89E-03
Molecular Mechanisms of Cancer	51	359	0.142		3.89E-03
Relaxin Signalling	24	132	0.182		4.17E-03

Cardiac ??-adrenergic Signalling	24	132	0.182	-0.243	4.17E-03
LPS-stimulated MAPK Signalling	16	73	0.219	-4	4.17E-03
Ephrin B Signalling	16	73	0.219	-1.89	4.17E-03
BMP Signalling pathway	16	74	0.216		4.79E-03
Role of NFAT in Regulation of the Immune Response	27	158	0.171	-3.578	5.01E-03
ERK/MAPK Signalling	30	183	0.164	-1.225	5.25E-03
ERK5 Signalling	14	61	0.23	-2.828	5.25E-03
PI3K/AKT Signalling	22	120	0.183	-0.853	5.62E-03
Amyloid Processing	12	50	0.24	-2.449	7.76E-03
Parkinson's Signalling	6	15	0.4		8.13E-03
Inhibition of Angiogenesis by TSP1	9	32	0.281	-2.646	9.33E-03

Supplementary Table S8. Gene set enrichment analysis (GSEA) for ageing with KEGG pathways. FDR q value (FDR, false discovery rate); FWER p value (FWER, family wise-error rate); ES (enrichment score) and NES (normalized enrichment score).

Supplementary Table S6. Gene set enrichment analysis for ageing						
NAME	SIZE	ES	NES	NOM p-val	FDR q-val	FWER p-val
Enrichment in Old						
RIBOSOME	76	0.49	5.01	0	0	0
UPDEGS_NOROS	35	0.67	4.73	0	0	0
UPDEGS_NFKB	35	0.55	3.82	0	0	0
COMPLEMENT_AND_COAGULATION_CASCADES	36	0.50	3.52	0	0	0
LEISHMANIA_INFECTION	46	0.40	3.23	0	0	0
CELL_ADHESION_MOLECULES_CAMS	89	0.29	3.17	0	0	0
ALLOGRAFT_REJECTION	22	0.55	3.05	0	0	0
GRAFT_VERSUS_HOST_DISEASE	23	0.53	2.99	2.01E-03	1.59E-04	0.001
SYSTEMIC_LUPUS_ERYTHEMATOSUS	51	0.34	2.84	0	1.42E-04	0.001
CYTOKINE_CYTOKINE_RECEPTOR_INTERACTION	110	0.22	2.61	0	1.07E-03	0.009
HEMATOPOIETIC_CELL_LINEAGE	34	0.37	2.57	0	1.62E-03	0.015
ASTHMA	13	0.57	2.48	0	1.80E-03	0.018
FOCAL_ADHESION	148	0.18	2.46	0	1.75E-03	0.019
TYPE_I_DIABETES_MELLITUS	29	0.37	2.42	0	2.14E-03	0.025
NOTCH_SIGNALLING_PATHWAY	37	0.34	2.42	0	2.23E-03	0.028
SMALL_CELL_LUNG_CANCER	61	0.26	2.41	0	2.09E-03	0.028
ECM_RECEPTOR_INTERACTION	51	0.28	2.35	2.01E-03	3.23E-03	0.046
AUTOIMMUNE_THYROID_DISEASE	29	0.36	2.34	0	3.40E-03	0.051
ANTIGEN_PROCESSING_AND_PRESENTATION	51	0.28	2.32	4.01E-03	3.66E-03	0.058
PROSTATE_CANCER	74	0.23	2.31	0	3.47E-03	0.058
CHRONIC_MYELOID_LEUKEMIA	62	0.24	2.26	0	5.03E-03	0.086
NATURAL_KILLER_CELL_MEDIATED_CYTOTOXICITY	83	0.20	2.20	0	7.14E-03	0.126
MAPK_SIGNALLING_PATHWAY	190	0.13	2.17	0	8.29E-03	0.152
LYSINE_DEGRADATION	36	0.30	2.14	0	9.98E-03	0.185
PPAR_SIGNALLING_PATHWAY	41	0.28	2.09	2.04E-03	1.27E-02	0.236
REGULATION_OF_ACTIN_CYTOSKELETON	146	0.15	2.09	6.00E-03	1.23E-02	0.238
INTESTINAL_IMMUNE_NETWORK_FOR_IGA_PRODUCTION	25	0.35	2.09	2.06E-03	1.23E-02	0.244
LEUKOCYTE_TRANSENDOTHELIAL_MIGRATION	74	0.20	2.01	7.71E-03	1.95E-02	0.372
VIRAL_MYOCARDITIS	52	0.23	1.94	1.02E-02	2.83E-02	0.502
FATTY_ACID_METABOLISM	33	0.28	1.90	8.42E-03	3.48E-02	0.592
PATHWAYS_IN_CANCER	228	0.11	1.87	6.25E-03	4.03E-02	0.654
TGF_BETA_SIGNALLING_PATHWAY	55	0.20	1.78	1.61E-02	6.21E-02	0.822
ADHERENS_JUNCTION	67	0.18	1.75	2.20E-02	7.10E-02	0.869
NON_SMALL_CELL_LUNG_CANCER	45	0.21	1.75	2.22E-02	6.95E-02	0.874
ARRHYTHMOGENIC_RIGHT_VENTRICULAR_CARDIOMYOPATHY_ARVC	56	0.19	1.69	2.47E-02	8.73E-02	0.933
B_CELL_RECEPTOR_SIGNALLING_PATHWAY	55	0.19	1.65	3.35E-02	1.02E-01	0.962
PANCREATIC_CANCER	58	0.19	1.65	2.85E-02	1.01E-01	0.963
TIGHT_JUNCTION	91	0.15	1.65	2.59E-02	1.01E-01	0.966
TRYPTOPHAN_METABOLISM	26	0.26	1.59	4.72E-02	1.31E-01	0.983

VALINE_LEUCINE_AND_ISOLEUCINE_DEGRADATION	40	0.21	1.54	5.40E-02	1.58E-01	0.998
Enrichment in Young						
NAME	SIZE	ES	NES	NOM p-val	FDR q-val	FWER p-val
OXIDATIVE_PHOSPHORYLATION	98	-0.42	-4.97	0	0	0
PARKINSONS_DISEASE	98	-0.40	-4.70	0	0	0
HUNTINGTONS_DISEASE	147	-0.27	-3.77	0	0	0
ALZHEIMERS_DISEASE	131	-0.27	-3.49	0	0	0
DNDEGS_MITODYS	36	-0.47	-3.37	0	0	0
DNDEGS_OXPHOS	24	-0.51	-2.95	0	0	0
NEUROACTIVE_LIGAND_RECEPTOR_INTERACTION	119	-0.21	-2.74	0	0	0
CARDIAC_MUSCLE_CONTRACTION	55	-0.30	-2.63	0	3.17E-04	0.003
PROTEASOME	39	-0.32	-2.37	0	3.17E-03	0.036
CALCIUM_SIGNALLING_PATHWAY	115	-0.18	-2.19	0	1.03E-02	0.123
OLFACTORY_TRANSDUCTION	36	-0.29	-2.10	2.02E-03	1.80E-02	0.218
AMINOACYL_TRNA_BIOSYNTHESIS	29	-0.32	-2.08	0	1.76E-02	0.229
REGULATION_OF_AUTOPHAGY	22	-0.37	-2.07	0	1.74E-02	0.243
RNA_POLYMERASE	27	-0.32	-2.01	4.24E-03	2.50E-02	0.356
EPITHELIAL_CELL_SIGNALLING_IN_HELICOBACTER_PYLORI_INFECTION	53	-0.22	-1.90	1.66E-02	4.67E-02	0.586
SPLICEOSOME	90	-0.17	-1.89	5.98E-03	4.50E-02	0.595
GLYCOSAMINOGLYCAN_BIOSYNTHESIS_HEPARAN_SULFATE	21	-0.32	-1.76	2.12E-02	8.58E-02	0.839
UBIQUITIN_MEDIATED_PROTEOLYSIS	106	-0.14	-1.71	2.10E-02	1.03E-01	0.897
RIBOFLAVIN_METABOLISM	11	-0.42	-1.66	2.98E-02	1.22E-01	0.939
GLYCOSPHINGOLIPID_BIOSYNTHESIS_LACTO_AND_NEOLACTO_SERIES	19	-0.30	-1.60	4.97E-02	1.56E-01	0.973

Supplementary Table S9. IPA upstream regulator analysis for LOAD

Supplementary Table S7. IPA upstream regulator analysis for LOAD					
Upstream Regulator	metaZscore	Molecule Type	Activation z-score	p-value of overlap	Bonferroni
Upstream Regulator for up regulated DGEs					
lipopolysaccharide		chemical drug	9.35	9.82E-59	1.91E-55
IFNG		cytokine	7.646	5.24E-50	1.02E-46
TNF		cytokine	9.099	1.14E-44	2.21E-41
TGFB1		growth factor	7.692	1.17E-43	2.27E-40
tretinoin		chemical - endogenous mammalian	8.228	1.20E-43	2.33E-40
dexamethasone		chemical drug	5.474	9.17E-36	1.78E-32
IL6		cytokine	6.526	1.11E-29	2.15E-26
TP53		transcription regulator	5.11	2.11E-28	4.09E-25
IL4		cytokine	4.672	5.53E-28	1.07E-24
IL1B		cytokine	7.236	6.16E-28	1.20E-24
CD40LG		cytokine	4.593	4.36E-25	8.46E-22
NFkB (complex)		complex	7.956	5.57E-24	1.08E-20
cisplatin		chemical drug	6.131	1.54E-23	2.99E-20
IL13		cytokine	3.105	3.74E-23	7.26E-20
CSF2		cytokine	5.89	5.22E-23	1.01E-19
poly rI:rC-RNA		chemical reagent	7.561	6.14E-23	1.19E-19
STAT3	7.203	transcription regulator	6.635	7.24E-23	1.40E-19
inosine		chemical - endogenous mammalian	5.231	5.47E-22	1.06E-18

phorbol myristate acetate		chemical drug	7.569	1.06E-21	2.06E-18
TLR4	4.861	transmembrane receptor	5.504	1.22E-21	2.37E-18
IL2		cytokine	4.284	1.80E-21	3.49E-18
camptothecin		chemical reagent	3.065	1.48E-20	2.87E-17
OSM		cytokine	5.305	2.11E-20	4.09E-17
NFKBIA	8.282	transcription regulator	2.419	1.41E-19	2.74E-16
E. coli B4 lipopolysaccharide		chemical toxicant	5.659	1.41E-19	2.74E-16
STAT1		transcription regulator	4.792	4.95E-19	9.60E-16
NR3C1		ligand-dependent nuclear receptor	3.069	6.98E-19	1.35E-15
beta-estradiol		chemical - endogenous mammalian	2.486	2.13E-18	4.13E-15
F2		peptidase	6.482	7.01E-18	1.36E-14
indomethacin		chemical drug	3.956	2.46E-17	4.77E-14
PDGF BB		complex	5.274	4.73E-17	9.18E-14
TGM2		enzyme	5.232	4.83E-17	9.37E-14
lomustine		chemical drug	4.572	1.48E-16	2.87E-13
triamcinolone acetonide		chemical drug	2.403	3.14E-16	6.09E-13
Interferon alpha		group	6.051	3.91E-16	7.59E-13
forskolin		chemical toxicant	4.631	4.03E-16	7.82E-13
E. coli B5 lipopolysaccharide		chemical - endogenous non-mammalian	5.859	1.01E-15	1.96E-12
hexachlorobenzene		chemical toxicant	4.888	1.26E-15	2.44E-12
gentamicin C		chemical drug	4.99	1.73E-15	3.36E-12
CSF3		cytokine	2.294	1.85E-15	3.59E-12
IGF1	-6.721	growth factor	3.585	2.05E-15	3.98E-12
fenamic acid		chemical reagent	4.706	2.10E-15	4.07E-12
2-bromoethylamine		chemical reagent	4.656	3.72E-15	7.22E-12
triamterene		chemical drug	4.954	6.29E-15	1.22E-11
IL15	7.395	cytokine	3.457	6.40E-15	1.24E-11
HGF	6.33	growth factor	6.005	8.36E-15	1.62E-11
vancomycin		chemical drug	4.782	9.73E-15	1.89E-11
IL1		group	5.27	1.06E-14	2.06E-11
prostaglandin E2		chemical - endogenous mammalian	3.279	1.36E-14	2.64E-11
TCR		complex	2.783	2.56E-14	4.97E-11
ERBB2		kinase	2.626	3.87E-14	7.51E-11
hydrogen peroxide		chemical - endogenous mammalian	5.876	4.21E-14	8.17E-11
MYD88	4.999	other	6.018	5.54E-14	1.07E-10
SP1	4.807	transcription regulator	5.769	6.06E-14	1.18E-10
EGF		growth factor	5.055	9.97E-14	1.93E-10
CSF1	5.575	cytokine	4.391	1.02E-13	1.98E-10
FSH		complex	2.384	1.18E-13	2.29E-10
SPI1		transcription regulator	4.962	1.22E-13	2.37E-10
TNFSF11		cytokine	4.808	1.28E-13	2.48E-10
thioacetamide		chemical toxicant	4.644	1.75E-13	3.40E-10
doxorubicin		chemical drug	5.309	1.94E-13	3.76E-10
phenacetin		chemical drug	4.338	2.39E-13	4.64E-10

benzyloxycarbonyl-Leu-Leu-Leu aldehyde		chemical - protease inhibitor	3.317	3.05E-13	5.92E-10
Ifn		group	3.716	3.76E-13	7.29E-10
allopurinol		chemical drug	4.702	4.58E-13	8.89E-10
Tgf beta		group	3.749	5.23E-13	1.01E-09
IRF1	6.649	transcription regulator	4.312	5.26E-13	1.02E-09
bleomycin		chemical drug	5.503	7.97E-13	1.55E-09
PI3K (complex)		complex	4.392	9.56E-13	1.85E-09
trichostatin A		chemical drug	4.147	1.29E-12	2.50E-09
APP		other	3.437	2.93E-12	5.68E-09
MYC	5.401	transcription regulator	2.112	3.78E-12	7.33E-09
CD40		transmembrane receptor	3.107	8.92E-12	1.73E-08
ethionine		chemical toxicant	4.123	9.24E-12	1.79E-08
paclitaxel		chemical drug	2.036	9.82E-12	1.91E-08
TNFSF10		cytokine	2.145	1.03E-11	2.00E-08
IL27		cytokine	4.123	1.11E-11	2.15E-08
Cg		complex	4.911	1.16E-11	2.25E-08
HIF1A		transcription regulator	4.855	1.16E-11	2.25E-08
RELA	6.205	transcription regulator	4.749	1.30E-11	2.52E-08
SMARCA4		transcription regulator	3.594	1.74E-11	3.38E-08
methylprednisolone		chemical drug	2.169	1.86E-11	3.61E-08
STAT4	-5.665	transcription regulator	6.108	3.04E-11	5.90E-08
TREM1		transmembrane receptor	2.86	3.14E-11	6.09E-08
IFNA2		cytokine	4.739	4.73E-11	9.18E-08
JUN		transcription regulator	3.981	6.27E-11	1.22E-07
CXCL12		cytokine	3.411	7.88E-11	1.53E-07
IL1A		cytokine	4.421	1.06E-10	2.06E-07
captopril		chemical drug	2.262	1.24E-10	2.41E-07
IKBKB	5.226	kinase	3.239	1.26E-10	2.44E-07
decitabine		chemical drug	6.16	1.30E-10	2.52E-07
arsenic trioxide		chemical drug	3.529	1.34E-10	2.60E-07
AGN194204		chemical drug	4.414	1.42E-10	2.75E-07
AKT1		kinase	2.358	1.47E-10	2.85E-07
IL12 (complex)		complex	4.071	1.48E-10	2.87E-07
TICAM1		other	5.255	1.56E-10	3.03E-07
NFKB1	6.131	transcription regulator	4.536	1.81E-10	3.51E-07
dihydrotestosterone		chemical - endogenous mammalian	4.382	1.95E-10	3.78E-07
lactacystin		chemical - protease inhibitor	2.847	2.04E-10	3.96E-07
ETS1		transcription regulator	3.815	2.66E-10	5.16E-07
LDL		complex	3.129	2.91E-10	5.65E-07
CEBPA		transcription regulator	3.34	3.32E-10	6.44E-07
phenylbutazone		chemical drug	4.321	4.11E-10	7.97E-07
CTNNB1		transcription regulator	4.842	4.97E-10	9.64E-07
ethanol		chemical - endogenous mammalian	3.755	5.69E-10	1.10E-06
calcitriol		chemical drug	2.277	7.45E-10	1.45E-06
PRL		cytokine	3.845	8.24E-10	1.60E-06
P38 MAPK		group	4.482	1.08E-09	2.10E-06

RETNLB		other	4.571	1.10E-09	2.13E-06
TGFA		growth factor	2.39	1.10E-09	2.13E-06
PGR		ligand-dependent nuclear receptor	4.038	1.32E-09	2.56E-06
resiquimod		chemical drug	3.402	1.35E-09	2.62E-06
cardiotoxin		chemical - other	5.196	1.55E-09	3.01E-06
SMAD4	5.82	transcription regulator	2.593	1.64E-09	3.18E-06
SYVN1		transporter	4.785	1.64E-09	3.18E-06
KITLG		growth factor	4.636	1.68E-09	3.26E-06
carbon tetrachloride		chemical toxicant	4.197	1.88E-09	3.65E-06
ERK		group	4.427	2.72E-09	5.28E-06
methotrexate		chemical drug	2.372	2.81E-09	5.45E-06
IL5		cytokine	5.233	3.11E-09	6.03E-06
AGT		growth factor	5.34	3.83E-09	7.43E-06
Vegf		group	6.824	5.22E-09	1.01E-05
CEBPB	7.844	transcription regulator	2.999	5.46E-09	1.06E-05
Ifnar		group	3.72	6.14E-09	1.19E-05
ERK1/2		group	3.884	6.85E-09	1.33E-05
GATA1		transcription regulator	2.035	6.94E-09	1.35E-05
CAMP		other	3.139	7.41E-09	1.44E-05
TLR3		transmembrane receptor	4.645	8.29E-09	1.61E-05
carboplatin		chemical drug	2.523	8.34E-09	1.62E-05
IRF8		transcription regulator	2.713	9.02E-09	1.75E-05
CCL5		cytokine	3.371	9.58E-09	1.86E-05
IRF7	6.129	transcription regulator	5.334	1.02E-08	1.98E-05
SELPLG	5.971	other	3.606	1.18E-08	2.29E-05
ERG		transcription regulator	3.956	1.28E-08	2.48E-05
TGFBR2	5.355	kinase	2.358	1.39E-08	2.70E-05
EGFR	5.637	kinase	2.753	1.80E-08	3.49E-05
Pam3-Cys-Ser-Lys4		chemical reagent	2.384	1.86E-08	3.61E-05
peptidoglycan		chemical - endogenous non-mammalian	3.366	2.28E-08	4.42E-05
SRC		kinase	2.266	2.43E-08	4.71E-05
RAF1	5.926	kinase	5.032	2.80E-08	5.43E-05
rosiglitazone		chemical drug	2.213	3.14E-08	6.09E-05
HRG		other	3.162	3.68E-08	7.14E-05
5-O-mycolyl-beta-araf-(1->2)-5-O-mycolyl-alpha-araf-(1->1')-glycerol		chemical - endogenous non-mammalian	4.472	4.26E-08	8.26E-05
TWIST1		transcription regulator	2.248	4.93E-08	9.56E-05
4-methylnitrosoamino-1-(3-pyridinyl)-1-butanone		chemical toxicant	2.989	5.49E-08	1.07E-04
MAP3K14		kinase	3.364	6.46E-08	1.25E-04
lipoteichoic acid		chemical - endogenous non-mammalian	3.282	6.46E-08	1.25E-04
alitretinoin		chemical drug	2.022	8.84E-08	1.71E-04
FGF2		growth factor	3.815	1.04E-07	2.02E-04
MAPKAPK2	7.373	kinase	2.743	1.16E-07	2.25E-04
cyclophosphamide		chemical drug	2.031	1.20E-07	2.33E-04
MAP2K1	-5.028	kinase	3.302	1.25E-07	2.43E-04

IL18	7.009	cytokine	3.385	1.28E-07	2.48E-04
SAMSN1	5.64	other	4.472	1.43E-07	2.77E-04
CpG oligonucleotide		chemical drug	4.473	1.52E-07	2.95E-04
D-glucose		chemical - endogenous mammalian	2.72	1.59E-07	3.08E-04
TET2		other	3.606	1.71E-07	3.32E-04
SELP		transmembrane receptor	3.464	1.82E-07	3.53E-04
Mapk		group	2.942	1.92E-07	3.72E-04
EGR1	-6.552	transcription regulator	3.906	2.41E-07	4.68E-04
mir-223		microrna	2.761	2.43E-07	4.71E-04
SASH1	5.975	other	4.123	3.32E-07	6.44E-04
IL3		cytokine	2.995	3.32E-07	6.44E-04
PRKCE		kinase	3.423	3.90E-07	7.57E-04
EDN1		cytokine	3.987	3.94E-07	7.64E-04
lipid A		chemical toxicant	3.44	4.43E-07	8.59E-04
SMARCB1		transcription regulator	3.845	5.73E-07	1.11E-03
EBI3	6.463	cytokine	2.84	5.96E-07	1.16E-03
RET		kinase	2.729	6.26E-07	1.21E-03
Tnf (family)		group	2.65	6.48E-07	1.26E-03
bucladesine		chemical toxicant	3.522	7.34E-07	1.42E-03
REL		transcription regulator	3.289	8.07E-07	1.57E-03
motexafin gadolinium		chemical drug	2.449	8.40E-07	1.63E-03
tunicamycin		chemical - endogenous non-mammalian	2.559	8.68E-07	1.68E-03
mycophenolic acid		chemical drug	2.714	8.86E-07	1.72E-03
MET		kinase	3.179	9.37E-07	1.82E-03
NRG1	-5.386	growth factor	2.42	1.02E-06	1.98E-03
thapsigargin		chemical toxicant	3.022	1.05E-06	2.04E-03
ETS2		transcription regulator	2.959	1.13E-06	2.19E-03
salmonella minnesota R595 lipopolysaccharides		chemical - endogenous non-mammalian	4.307	1.14E-06	2.21E-03
IL17A		cytokine	4.532	1.30E-06	2.52E-03
CD44	5.738	enzyme	2.676	1.34E-06	2.60E-03
TYROBP	7.113	transmembrane receptor	2.213	1.35E-06	2.62E-03
TNFSF12		cytokine	3.957	1.38E-06	2.68E-03
TLR2	6.847	transmembrane receptor	3.453	1.48E-06	2.87E-03
17-alpha-ethinylestradiol		chemical drug	2.037	1.48E-06	2.87E-03
PTGS2		enzyme	3.364	1.68E-06	3.26E-03
Akt		group	2.14	1.87E-06	3.63E-03
JAK2		kinase	3.22	1.89E-06	3.67E-03
ATM		kinase	2.105	2.06E-06	4.00E-03
DETA-NONOate		chemical reagent	2.206	2.31E-06	4.48E-03
AR		ligand-dependent nuclear receptor	2.607	2.54E-06	4.93E-03
tributyrin		chemical drug	3.359	2.67E-06	5.18E-03
MTPN		transcription regulator	4.067	2.87E-06	5.57E-03
hyaluronic acid		chemical - endogenous mammalian	2.045	3.04E-06	5.90E-03
Insulin		group	2.29	3.07E-06	5.96E-03

E. coli lipopolysaccharide		chemical - endogenous non-mammalian	3.175	3.28E-06	6.36E-03
TLR7	6.476	transmembrane receptor	3.917	3.97E-06	7.70E-03
etoposide		chemical drug	3.581	4.00E-06	7.76E-03
vitamin K2		chemical drug	2.8	4.54E-06	8.81E-03
5-azacytidine		chemical drug	4.115	4.88E-06	9.47E-03
EIF2AK2		kinase	2.48	5.69E-06	1.10E-02
LIF		cytokine	3.42	5.75E-06	1.12E-02
Ifn gamma		complex	2.517	6.71E-06	1.30E-02
dimethyl sulfoxide		chemical drug	2.848	7.24E-06	1.40E-02
bromodeoxyuridine		chemical drug	3.544	7.48E-06	1.45E-02
CXCR4	6.322	g-protein coupled receptor	3.26	7.48E-06	1.45E-02
NOS2		enzyme	3.971	7.55E-06	1.46E-02
PTK2		kinase	2.398	8.72E-06	1.69E-02
IFN alpha/beta		group	3.478	9.11E-06	1.77E-02
TGFB2	4.843	growth factor	2.236	9.23E-06	1.79E-02
EPAS1		transcription regulator	2.905	1.10E-05	2.13E-02
hemozoin		chemical - endogenous non-mammalian	2.946	1.10E-05	2.13E-02
GC-GCR dimer		complex	2.621	1.36E-05	2.64E-02
PRKCD		kinase	2.177	1.46E-05	2.83E-02
7-ethyl-10-hydroxy-camptothecin		chemical drug	2.414	1.67E-05	3.24E-02
VEGFA		growth factor	4.577	1.93E-05	3.74E-02
cobalt chloride		chemical reagent	2.998	1.94E-05	3.76E-02
GLI1		transcription regulator	4.504	1.98E-05	3.84E-02
TP63		transcription regulator	2.982	2.05E-05	3.98E-02
pirinixic acid		chemical toxicant	2.941	2.06E-05	4.00E-02
arsenite		chemical toxicant	2.299	2.11E-05	4.09E-02
Gm-csf		group	2.239	2.14E-05	4.15E-02
KLF5		transcription regulator	2.117	2.14E-05	4.15E-02
NEDD9		other	3.162	2.20E-05	4.27E-02
Pdgf (complex)		complex	3.091	2.26E-05	4.38E-02
trovafloxacin		chemical drug	3.207	2.31E-05	4.48E-02
reactive oxygen species		chemical toxicant	3.832	2.48E-05	4.81E-02
TNFRSF1A	7.527	transmembrane receptor	2.421	2.48E-05	4.81E-02
Hbb-b1		transporter	2.335	2.56E-05	4.97E-02

Upstream Regulator for down regulated DGEs

Upstream Regulator	metaZscore	Molecule Type	Activation z-score	p-value of overlap	Boferroni
REST		transcription regulator	3.024	8.22E-09	1.53E-06
RICTOR		other	6	2.52E-05	4.69E-03

Supplementary Table S10. List of 36 target DEGs of RICTOR in LOAD.

Entrez.Gene	Symbol	metaZscore	metaPval	effect	Bonferroni	BraakR	atrophyR
4711	NDUFB5	-7.70	1.40E-14	------	3.29E-10	-0.67	-0.52
4701	NDUFA7	-6.94	3.90E-12	--?--+	9.17E-08	-0.66	-0.56
516	ATP5G1	-6.66	2.69E-11	--?---	6.34E-07	-0.70	-0.60

51382	ATP6V1D	-6.62	3.70E-11	------	8.70E-07	-0.64	-0.53
4704	NDUFA9	-6.58	4.86E-11	------	1.14E-06	-0.67	-0.50
1355	COX15	-6.52	6.94E-11	--?---	1.63E-06	-0.61	-0.47
4723	NDUFV1	-6.30	2.91E-10	-----+	6.84E-06	-0.67	-0.53
1537	CYC1	-6.03	1.65E-09	--?---	3.89E-05	-0.67	-0.55
522	ATP5J	-5.93	2.95E-09	--?---	6.94E-05	-0.69	-0.54
5702	PSMC3	-5.88	4.08E-09	--?---	9.61E-05	-0.68	-0.55
5714	PSMD8	-5.69	1.31E-08	------	3.07E-04	-0.61	-0.47
7385	UQCRC2	-5.64	1.72E-08	--?--+	4.05E-04	-0.65	-0.53
529	ATP6V1E1	-5.60	2.12E-08	--?---	4.99E-04	-0.66	-0.56
518	ATP5G3	-5.54	3.02E-08	--?---	7.12E-04	-0.65	-0.52
509	ATP5C1	-5.47	4.53E-08	--+---	1.06E-03	-0.64	-0.52
9167	COX7A2L	-5.45	5.17E-08	--?--+	1.22E-03		-0.46
5719	PSMD13	-5.41	6.36E-08	--+---	1.50E-03		-0.49
51187	RSL24D1	-5.37	7.70E-08	------	1.81E-03		
515	ATP5F1	-5.37	8.09E-08	--?---	1.90E-03	-0.61	-0.48
534	ATP6V1G2	-5.35	8.85E-08	------	2.08E-03	-0.66	-0.57
27089	UQCRQ	-5.35	8.86E-08	--?--+	2.08E-03	-0.73	-0.57
6171	RPL41	-5.23	1.66E-07	--?--+	3.90E-03	-0.70	-0.52
4700	NDUFA6	-5.22	1.82E-07	--?---	4.29E-03	-0.65	-0.53
4712	NDUFB6	-5.20	1.96E-07	-+?---	4.60E-03	-0.63	-0.50
5718	PSMD12	-5.19	2.10E-07	-+?---	4.94E-03		-0.49
5686	PSMA5	-5.18	2.24E-07	------	5.27E-03	-0.66	-0.55
10632	ATP5L	-5.14	2.74E-07	--?---	6.46E-03	-0.66	-0.52
5695	PSMB7	-5.14	2.80E-07	------	6.59E-03	-0.65	-0.51
4722	NDUFS3	-5.00	5.63E-07	------	1.32E-02	-0.67	-0.53
51606	ATP6V1H	-4.96	7.10E-07	--?---	1.67E-02	-0.61	-0.52
523	ATP6V1A	-4.96	7.10E-07	------	1.67E-02		-0.45
4705	NDUFA10	-4.94	7.74E-07	-----+	1.82E-02		-0.45
1327	COX4I1	-4.90	9.42E-07	--+---	2.22E-02	-0.70	-0.58
526	ATP6V1B2	-4.88	1.06E-06	--?---	2.50E-02	-0.65	-0.51
4706	NDUFAB1	-4.80	1.62E-06	------	3.82E-02	-0.65	-0.51
1347	COX7A2	-4.74	2.12E-06	-+----	4.99E-02	-0.66	-0.54

Supplementary Table S11. IPA upstream regulator analysis for ageing

Supplementary Table S9. IPA upstream regulator analysis for ageing					
Upstream Regulator	Log Ratio	Molecule Type	Activation z-score	p-value of overlap	Bonferroni
Upstream Regulator for up regulated DEGs					
MYCN	0.143	transcription regulator	3.030	4.65E-15	3.18E-12
HRAS	-0.055	enzyme	2.332	3.92E-12	2.68E-09
tretinoin		chemical - endogenous mammalian	5.225	3.96E-11	2.71E-08
TGFB1	0.839	growth factor	4.541	4.33E-11	2.96E-08
TP53	0.217	transcription regulator	5.590	6.96E-11	4.76E-08
dexamethasone		chemical drug	5.394	6.22E-10	4.25E-07
dihydrotestosterone		chemical - endogenous mammalian	3.112	2.29E-09	1.57E-06
cisplatin		chemical drug	3.809	1.27E-08	8.69E-06
MITF	0.332	transcription regulator	5.237	8.82E-08	6.03E-05
JUN	-0.490	transcription regulator	2.598	9.97E-08	6.82E-05
AKT1	0.225	kinase	2.454	3.01E-07	2.06E-04
SYVN1	0.193	transporter	4.574	3.05E-07	2.09E-04
motexafin gadolinium		chemical drug	2.449	4.42E-07	3.02E-04
SMARCA4	-0.378	transcription regulator	3.520	5.81E-07	3.97E-04
triamcinolone acetonide		chemical drug	2.744	1.88E-06	1.29E-03
IFNG		cytokine	2.503	2.79E-06	1.91E-03
PGR	0.055	ligand-dependent nuclear receptor	4.352	5.54E-06	3.79E-03
progesterone		chemical - endogenous mammalian	4.791	5.70E-06	3.90E-03
FSH		complex	2.454	5.77E-06	3.95E-03
FN1	0.481	enzyme	3.393	2.39E-05	1.63E-02
PDGF BB		complex	4.439	2.46E-05	1.68E-02

Upstream Regulator	Log Ratio	Molecule Type	Activation z-score	p-value of overlap	Bonferroni
NR3C1	-0.521	ligand-dependent nuclear receptor	2.517	2.52E-05	1.72E-02
MYOD1		transcription regulator	3.739	2.61E-05	1.79E-02
ERK		group	2.327	3.80E-05	2.60E-02
LIMS1	0.457	other	2.224	5.00E-05	3.42E-02
Insulin		group	3.386	6.01E-05	4.11E-02
Up stream regulators for down regulated DEGs					
Upstream Regulator	Log Ratio	Molecule Type	Activation z-score	p-value of overlap	Bonferroni
RICTOR	0.459	other	6.487	3.24E-08	6.35E-06
CD 437		chemical drug	6.164	4.17E-06	8.17E-04
REST	0.410	transcription regulator	3.098	1.42E-05	2.78E-03
topotecan		chemical drug	2.501	2.11E-04	4.14E-02

Supplementary Table S12. Number of neighbours for the top DEGs in PPI DEGs network. It contains the number of first neighbours, number of DEG, number of up/down regulated DEGs among the first neighbours (see Supplementary Fig. S4)

TopDEGs	Neighbours	DEGs	up DEGs	down DEGs
NFKBIA	64	23	13	10
C1QA	43	10	9	1
TRIP10	12	6	4	2
BDNF	7	1	1	0
DUSP4	7	1	0	1
DOK3	5	4	4	0
SEMA3F	4	0	0	0
KCNF1	4	1	0	1
ITPKB	3	1	0	1
CCKBR	3	1	0	1
CRH	2	0	0	0
LATS2	2	1	1	0
NUPR1	2	0	0	0
ZCCHC17	2	0	0	0
PPEF1	1	0	0	0
HIST1H2BD	1	0	0	0

Supplementary Table S13. Number of neighbours for GWAS hits in the PPI GWAS network. The able shows the number of first neighbours, number of DEGs, and number of up/down regulated DEGs among the first neighbours (see Supplementary Fig. S5)

GWASAD	Neighbours	DEGs	up DEGs	down DEGs
APOE	124	18	10	8
PTK2B	55	16	13	3
CLU	44	9	7	2
BIN1	32	7	4	3
INPP5D	22	7	7	0
CD2AP	19	3	3	0
MEF2C	19	5	4	1
TRIP4	14	6	5	1
CR1	9	6	6	0
PICALM	7	1	1	0
CELF1	4	0	0	0
SORL1	3	2	2	0
FERMT2	1	0	0	0
TREM2	1	1	1	0

Methods

Data collection and pre-processing

We searched arrayExpress (https://www.ebi.ac.uk/arrayexpress/) and GEO (http://www.ncbi.nlm.nih.gov/geo/) for all late-onset Alzheimer's disease (LOAD) related mRNA expression studies that included human post mortem brain tissues from super frontal gyrus (SFG) or prefrontal cortex (PFC), both of which are part of the brain frontal lobe. We found and downloaded six profile datasets with GEO accession number of GSE5281, GSE48350, GSE36980, GSE15222, GSE44770 and GSE33000. The GSE5281 and GSE48350 datasets, generated from the Affymatrix HG-U133-plus2 platform (GEO platform ID: GPL570), contained 54675 probesets and have 161 and 252 profiles respectively. We worked on the raw CEL files and completed the normalization using the RMA (Robust Multi-array Average) method implemented in the affy[2] R package (http://www.bioconductor.org). Pre-filtering was completed by allowing no more than 10% absent (present/absent call by affy mas5 algorithm) across all samples in each study and probesets with average expression level lower than 3 (after log2 transformation) were discarded afterwards. The latest version of probesets annotation file HG-U133_Plus_2.na34.annot.csv was downloaded from Affymetrix NetAffx Analysis Centre (www.affymetrix.com/analysis/index.affx). GSE36980 was generated from the Affymatrix Human Gene 1.0 ST platform (GEO platform ID: GPL6244) with 79 profiles. As with the above datasets, we worked on the raw CEL files to complete transcript level RMA normalization and excluded transcripts with average expression lower than 3. For this dataset, we also carried out probe level present/absent calls using the oligo[3] R package, i.e., we marked all probes having fewer than 10% absents across samples as good probes and discarded any transcripts that did not contain any good probes. Microarray annotation database package hugene10sttranscriptcluster.db in R was used for annotating this dataset. GSE44770 and GSE33000 were both generated from the Rosetta/Merck Human 44k microarray platform (GEO platform ID: GPL4372), containing 39302 probesets and including 230 and 623 case-control profiles respectively. We worked on the processed data without any pre-filtering. GSE15222 was generated from the

Illumina microarray platform (GEO platform ID: GPL2700) and contains 24354 probesets and 363 profiles. We worked on a sub-dataset containing 8650 pre-filtered probesets downloaded from Dr Myer's lab (http://labs.med.miami.edu/myers). In this data file, probesets had null values if their relevant detection scores were less than 0.99, and probesets with more than 30% null values were excluded. GSE44770 and GSE33000 contained only PFC tissue; GSE5281, GSE48350, GSE15222 and GSE36980 had multiple tissues. Alzheimer's disease is age-related, so in order to reduce the analysis bias induced by age we only included samples aged between 65 and 95 years old, and discarded any samples without age information. In this study, we only focused on PFC types because we were able to obtain the maximum number of gene expression profiles in this brain region, giving us a total number of 212 controls and 450 cases after age controlling.

We mapped probesets to Entrez Gene IDs (as gene IDs) in each study first, and removed probesets having multiple mapped genes. Furthermore, if a gene has multiple relevant probesets, then the probesets having the biggest absolute estimated effect size would be kept and the rest discarded. Supplementary Table S1 lists the numbers of case and control samples, and the number of unique genes in each study used for the meta-analysis.

We downloaded GSE53890 from GEO which contains 12 young (age < 40 yrs), 17 old (aged between 65 to 95) and other aged gene expression profiles in raw CEL file format generated from the Affymatrix HG-U133-plus2 platform. Similar data processing was completed for this non-AD cohort. After pre-processing, 25264 probesets were left. The limma[4] R package was used for detecting differentially expressed genes (DEGs) with Benjamini-Hochberg adjusted p-value < 0.01 and expression intensity > 5 (in log2 space).

GSE5281 contains six brain regions: entorhinal cortex (EC); hippocampus (HIP); medial temporal gyrus (MTG); posterior cingulate (PC); superior frontal gyrus (SFG) and primary visual cortex (PVC). DEGs in each region were identified by limma with the same criteria as above, we mapped the top meta-analysis DEGs to this dataset (see Supplementary Table S4).

References

1 Zhang, B. *et al.* Integrated systems approach identifies genetic nodes and networks in late-onset Alzheimer's disease. *Cell* **153**, 707-720, doi:10.1016/j.cell.2013.03.030 (2013).

2 Gautier, L., Cope, L., Bolstad, B. M. & Irizarry, R. A. affy--analysis of Affymetrix GeneChip data at the probe level. *Bioinformatics* **20**, 307-315, doi:10.1093/bioinformatics/btg405 (2004).

3 Carvalho, B. S. & Irizarry, R. A. A framework for oligonucleotide microarray preprocessing. *Bioinformatics* **26**, 2363-2367, doi:10.1093/bioinformatics/btq431 (2010).

4 GK, S. *Limma: linear models for microarray data*. 397-420 (Springer, 2005).
